# Endothelial Matrin‐3 Limits Cellular Senescence and Lipid Dysregulation to Protect Against Murine Atherosclerosis

**DOI:** 10.1096/fj.202602809RR

**Published:** 2026-10-17

**Authors:** Xiao Cheng, Fatema Yeasmin Tanni, Rachelle Nelson Zoni, Elham Abdollahi, Eli Childs, Xinghui Sun

**Affiliations:** ^1^ Department of Biochemistry University of Nebraska‐Lincoln Lincoln Nebraska USA; ^2^ Nebraska Center for the Prevention of Obesity Diseases Through Dietary Molecules University of Nebraska‐Lincoln Lincoln Nebraska USA

**Keywords:** atherosclerosis, cellular senescence, Matrin‐3, vascular endothelium

## Abstract

Matrin‐3 is a DNA‐ and RNA‐binding protein implicated in cardiovascular development. However, the role of endothelial Matrin‐3 in atherosclerosis has not been previously studied. Here, we found that Matrin‐3 silencing induces endothelial senescence, characterized by reduced cell proliferation, increased senescence‐associated β‐galactosidase activity, and impaired mitochondrial function in vitro. Quantitative proteomics and bulk RNA‐sequencing identified hundreds of differentially expressed genes and proteins in human endothelial cells. Bioinformatic analyses revealed enrichment of pathways related to DNA replication, p53 signaling, cell‐cycle regulation, and cellular senescence. To examine the role of endothelial Matrin‐3 in atherosclerosis, we generated Matrin‐3 floxed mice on a low‐density lipoprotein receptor‐knockout background and crossed them with mice carrying an endothelial cell‐specific, tamoxifen‐inducible Cre driver. In a mouse model of atherosclerosis, endothelial cell‐specific Matrin‐3 deficiency increased atherosclerotic lesion area by 1.41‐fold and was associated with increased plasma low‐density lipoprotein cholesterol and triglycerides, increased hepatic cholesterol and triglycerides, and substantial transcriptional alterations in the aortic intima. Endothelial Matrin‐3 deficiency also induced hepatic steatosis, fibrosis, inflammation, and endothelial senescence. Single‐nucleus RNA‐sequencing of the liver further revealed transcriptional reprogramming of hepatocytes, including alterations in pathways related to lipid metabolism, inflammation, and constitutive androstane receptor signaling, consistent with a functional link between endothelial Matrin‐3 deficiency and hepatocyte transcriptional responses. Finally, Matrin‐3 expression was reduced in mouse and human atherosclerotic plaques. Together, these findings identify endothelial Matrin‐3 as an important regulator of endothelial senescence and lipid homeostasis and demonstrate that endothelial Matrin‐3 deficiency is associated with both local vascular alterations and systemic metabolic dysfunction in atherosclerosis.

## Graphical abstract

Endothelial Matrin‐3 suppresses cellular senescence and protects against atherosclerosis. Matrin‐3 deficiency induces endothelial senescence, mitochondrial dysfunction, and activation of p53‐, cell cycle‐, and senescence‐related pathways. In atherosclerosis‐prone mice, endothelial Matrin‐3 deletion promotes atherosclerotic lesion formation and disrupts systemic and hepatic lipid homeostasis, accompanied by liver steatosis, fibrosis, inflammation, and senescence. Reduced Matrin‐3 expression in mouse and human atherosclerotic plaques further supports its protective role in vascular and metabolic homeostasis.

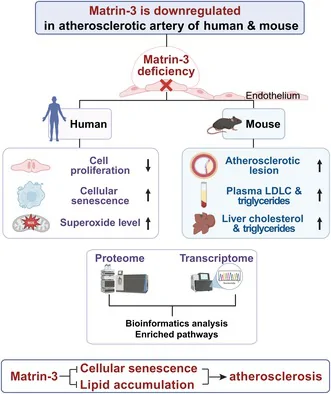

## 1 Introduction

Atherosclerosis is the leading cause of cardiovascular disease. Its initiation and progression involve multiple interconnected mechanisms, including low‐density lipoprotein accumulation and oxidation in the arterial intima, immune and inflammatory responses, defective efferocytosis, vascular smooth muscle cell dysregulation, extracellular matrix remodeling, and cellular senescence [1, 2, 3, 4]. Senescent vascular cells accumulate in human atherosclerotic lesions and exhibit multiple features of cellular dysfunction [5]. Studies in animal models have provided causal evidence that senescent vascular cells contribute to chronic inflammation, immune dysfunction, impaired cholesterol handling, atheroma formation, and plaque instability [6, 7, 8]. In particular, endothelial senescence can promote atherosclerosis through endothelial dysfunction, vascular inflammation, increased lipid entry into the vessel wall, and plaque progression [9, 10, 11]. However, the molecular mechanisms that regulate endothelial senescence and the relationships among endothelial dysfunction, local vascular alterations, and systemic metabolic abnormalities remain incompletely understood.

RNA‐binding proteins are important regulators of endothelial gene expression and cellular responses to metabolic and inflammatory stress and have increasingly been implicated in atherosclerosis [12]. For example, the RNA‐binding protein Quaking promotes monocyte differentiation into pro‐atherogenic macrophages by regulating pre‐mRNA splicing and gene expression [13]. Human antigen R binds to the 3′‐untranslated region of cathepsin S mRNA following adenosine‐to‐inosine RNA editing, thereby stabilizing the transcript and increasing its abundance [14]. Increased cathepsin S expression can promote atherosclerosis by degrading elastin and collagen and contributing to vascular inflammation and plaque instability [15, 16, 17]. RNA‐binding proteins can also regulate cellular functions relevant to atherosclerosis through interactions with long noncoding RNAs [18]. Together, these studies highlight RNA‐binding proteins as important regulators of vascular homeostasis and potential therapeutic targets for vascular disease [19].

Matrin‐3 is a nuclear protein that binds both DNA and RNA. Genetic variants in *MATR3* are associated with amyotrophic lateral sclerosis [20, 21], and altered Matrin‐3 expression or function has been implicated in several human pathological conditions [20, 21, 22, 23, 24, 25]. Disruption of Matrin‐3 in humans and mice is associated with cardiovascular abnormalities, including bicuspid aortic valve, aortic coarctation, and patent ductus arteriosus [25]. We previously identified Matrin‐3 as a protein‐binding partner of maternally expressed gene 3 (Meg3), a long noncoding RNA that regulates the DNA damage response and limits cellular senescence and obesity‐induced insulin resistance [26, 27]. We also demonstrated that Matrin‐3 constrains hepatic steatosis and cellular stress responses and delays the development of metabolic dysfunction‐associated steatotic liver disease [28]. In addition, a recent study showed that Matrin‐3 in macrophages suppresses inflammatory responses and limits atherosclerosis development [29]. However, whether endothelial Matrin‐3 regulates cellular senescence and atherosclerosis remains unknown.

In this study, we investigated the role of endothelial Matrin‐3 in cellular senescence and atherosclerosis. We found that Matrin‐3 limits cellular senescence in human endothelial cells and regulates hundreds of genes and proteins involved in DNA damage responses, cell‐cycle regulation, p53 signaling, and cellular senescence. Endothelial cell‐specific Matrin‐3 deficiency accelerated atherosclerosis and was associated with substantial transcriptional alterations in the aortic intima, consistent with a local vascular contribution to the phenotype. Unexpectedly, endothelial Matrin‐3 deficiency is also associated with hepatic lipid accumulation and increased circulating cholesterol and triglycerides. Single‐nucleus RNA‐sequencing of the liver revealed transcriptional reprogramming of hepatocytes, including alterations in pathways related to lipid metabolism, inflammation, and constitutive androstane receptor signaling, consistent with a functional link between endothelial Matrin‐3 deficiency and hepatocyte transcriptional responses. Thus, our findings demonstrate that endothelial Matrin‐3 deficiency is associated with both local vascular alterations and systemic metabolic dysfunction in the setting of accelerated atherosclerosis. Finally, reduced Matrin‐3 expression in human atherosclerotic arteries and aortic valves supports the potential relevance of these findings to human vascular disease.

## 2 Materials and Methods

### 2.1 Animals and Study Approval

Matrin‐3 floxed mice were generated as described in our previous studies [28]. Matrin‐3 floxed mice were bred with *Ldlr* ^−/−^ mice (strain #: 002207 from the Jackson Laboratory) and Cdh5(PAC)‐CreERT2 mice for several rounds to get two groups of mice were generated: *Matr3* ^fl/fl^;*Ldlr* ^−/−^ mice (hereafter, referred to as fl/fl) and *Matr3* ^fl/fl^;*Cre* ^+/−^;*Ldlr* ^−/−^ mice (iECKO for tamoxifen‐inducible endothelial cell‐specific Matrin‐3 knockout mice). Cdh5(PAC)‐CreERT2 mice were developed in the laboratory of Dr. Ralf Adams at the London Research Institute [30]. Mice received tamoxifen (*i.p*., 50 mg/kg/day; Sigma cat# T5648) for 5 days at 6 weeks of age. A week later, mice were fed a high‐fat diet (Harlan TD.88137, 42% calories from fat) with 18.9 g/L glucose and 23.1 g/L fructose added to the drinking water for 16 weeks to induce atherosclerosis.

All procedures and animal use were approved by the Institutional Animal Care and Use Committee at the University of Nebraska—Lincoln and in accordance with guidelines of the National Institutes of Health. All animals were purchased from the Institution Animal Care and Use Committee approved vendors and inbred in an American Association for Accreditation of Laboratory Animal Care‐approved animal facility.

### 2.2 Cell Culture

HUVECs (Lonza, cat# CC‐2519) and human coronary aortic endothelial cells (HCAECs) (Lonza, cat# CC‐2585) were cultured in endothelial cell growth medium EGM‐2 (Lonza, cat# CC‐3162) according to the manufacturer's instructions.

### 2.3 Lentiviral shRNA Cloning and Virus Production

Control shRNA (Ctl shRNA) oligos or Matrin‐3 shRNA (Matr3 shRNA) were cloned into pLKO.1‐TRC vector between AgeI and EcoRI sites. Lentivirus was produced in 293 T cells cultured in DMEM supplemented with 10% FBS, 1% PS, and 2 mM L‐glutamine. Cells from two confluent 15‐cm dishes were seeded into 12 poly‐L‐lysine‐coated 15‐cm dishes using 20 mL of medium per dish and incubated overnight at 37°C. At 80%–90% confluence, cells were transfected with PEI (Polysciences, cat# 23966) using a plasmid mix of pMD2.G, psPAX2, and lentiviral vector. Medium was replaced 16‐20 h post‐transfection. Supernatants were collected at 48 h and 72 h post‐transfection, pooled, and clarified by centrifugation at 783 × g for 5 min, followed by filtration through a 0.45‐μm filter. Viruses were concentrated by ultracentrifugation at 80000 × g for 2 h at 4°C using an SW32Ti rotor. The pellet was resuspended in 200 μL of 1 × HBSS, and the virus was aliquoted and stored at −80°C. The sequences of shRNA oligos were as follows: Ctl shRNA sense, 5′‐CCGGCAACAAGATGAAGAGCACCAACTCGAGTTGGTGCTCTTCATCTTGTTGTTTTTG‐3′; antisense, 5′‐AATTCAAAAACAACAAGATGAAGAGCACCAACTCGAGTTGGTGCTCTTCATCTTGTTG‐3′, Matr3 shRNA‐1 sense, 5′‐CCGGGCACTTTAGAAGAGATAGTTTCTCGAGAAACTATCTCTTCTAAAGTGCTTTTTG‐3′; antisense, 5′‐AATTCAAAAAGCACTTTAGAAGAGATAGTTTCTCGAGAAACTATCTCTTCTAAAGTGC‐3′, and Matr3 shRNA‐2 sense, 5′‐CCGGGAGACCGATCTTGCTAATTTACTCGAGTAAATTAGCAAGATCGGTCTCTTTTTG‐3′; antisense, 5′‐AATTCAAAAAGAGACCGATCTTGCTAATTTACTCGAGTAAATTAGCAAGATCGGTCTC‐3′.

### 2.4 EdU Incorporation and Staining and Quantification

HUVECs were seeded in 12‐well plates at a density of 50 000 cells per well and cultured for 24 h before lentiviral infection with control shRNA or Matrin‐3 shRNA. Twenty‐four hours after infection, the cells were replated at 70 000 cells per well and incubated for another 12 h at 37°C in a humidified incubator with 5% CO_2_.

Cell proliferation was assessed via EdU labeling using the Click‐iT EdU Alexa Fluor 488 Imaging Kit (Invitrogen, cat# C10337). The original culture medium was replaced with fresh medium supplemented with 10 μM EdU, and the cells were incubated for 4 h under the same conditions. Thereafter, cell fixation, permeabilization, EdU detection, and DNA staining were performed following the manufacturer's protocols. Fluorescence images of the stained cells were captured using an EVOS fluorescence microscope at 10× magnification. For quantitative analysis, 8–12 random fields were captured per condition, and EdU‐positive cells were counted using ImageJ software.

### 2.5 Senescence‐Associated β‐Galactosidase Staining and Quantification

HUVECs were seeded into 6‐well plates (2 wells per condition) at 100 000 cells/mL and infected with ctl shRNA or Matr3 shRNA the next day. After 16 h, the medium was replaced and then changed every 2 days. On Day 5 after infection, cells were plated into 12‐well plates at 90000 cells per well. After 24 h, β‐galactosidase staining was performed using a Senescence β‐galactosidase Staining Kit according to the manufacturer's instructions (Cell Signaling Technology, cat# 9860). Twelve random fields were captured per condition, and β‐gal‐positive cells were counted using ImageJ software.

### 2.6 MitoSOX Staining and Flow Cytometry

To measure mitochondrial superoxide production, HUVECs were transduced with lenti‐ctl shRNA or lenti‐matr3 shRNAs. After 40 h, cells were stained with MitoSOX Red superoxide radical‐specific reagent as described in our previous study [27]. After staining, MitoSOX was excited with a 488 nm laser, and a Texas red filter (610/40 nm) was used to detect the emission by flow cytometry.

### 2.7 Quantitative Proteomics and Data Analysis

Proteomic analysis using TMT10‐plex labeling and liquid chromatography–tandem mass spectrometry was performed as described in our previous study [26]. Briefly, HUVECs were transduced with lenti‐ctl shRNA or lenti‐matr3 shRNAs. After 40 h, cells were washed and scraped into ice‐cold 1 × PBS and spun at 300×*g* for 5 min. Proteins were prepared for digestion with trypsin. The peptides were labeled using the TMT10‐plex reagent, combined, and fractionated. Mass spectrometric analysis of each fraction was carried out using a Dionex U3000 nanoRSLC running a 2 h gradient through a 0.075 mm × 250 mm C18 Waters CSH130 peptide column feeding into a Q‐Exactive HF mass spectrometer (ThermoScientific). The protein identification and quantification were processed as described [26]. Gene symbols of the identified proteins and their relative expression were used for Enrichr analysis.

### 2.8 Bulk RNA‐Seq Sample Preparation, Sequencing, and Data Analysis

HCAEC cells obtained from Lonza were plated into 12‐well plates at 100 000 cells/well. Then, cells were infected with lentivirus expressing Ctl shRNA or Matr3 shRNA 24 h later. The medium was replaced at 16 h post‐infection, and cells were collected 40 h after viral infection. Total RNA extraction from HCAEC cells was performed using TRIzol reagent according to the manufacturer's instructions (Invitrogen, cat# 15596018). Total RNAs were sent to LC Sciences for RNA‐sequencing and standard data analysis. Briefly, poly(A) RNA sequencing library was prepared following Illumina's TruSeq‐stranded‐mRNA sample preparation protocol. The reads that contained adaptor contamination, low‐quality bases and undetermined bases were removed. HISAT2 was used to map reads to the Ensembl human genome GRch38 (release‐104). Differentially expressed genes were identified using DEseq2 in R.

Matrin‐3 fl/fl and iECKO mice were administered with tamoxifen as described above and fed on the high‐cholesterol diet to induce atherosclerosis. The thoracic aorta of each mouse was collected. Isolation of intimal RNA from aorta was performed as previously described in [31, 32]. Briefly, each aorta was carefully flushed with 1× dPBS, followed by intima peeling using TRIzol reagent. TRIzol was flushed for 10 s—10 s pause—another 10 s and collected in an Eppendorf tube (~300 μL total) and snap‐frozen with dry ice. Total RNA was extracted from using TRIzol reagent according to the manufacturer's instructions. RNA‐seq analysis was performed by Novogene. Briefly, the directional mRNA libraries were prepared following poly(A) enrichment. Libraries were sequenced on the NovaSeq platform using paired‐end 150 bp chemistry. The raw reads were filtered to remove low‐quality reads and adapter sequence. The filtered reads were aligned to the reference genome (mm10) using HISAT2. DESeq2 was used to identify differentially expressed genes in R. The gene symbols of differentially expressed genes were used to identify enriched pathways using Enrichr [33]. Plots were generated using ggplot2 (4.0.1) package in R (4.4.3).

### 2.9 Single‐Nucleus RNA‐Seq and Data Analysis

Sequencing libraries were prepared using the 10× Genomics GEM‐X Universal 3′ Gene Expression v4 4‐plex Reagent Kits at Single‐Cell Genomics Core Facility of the University of Nebraska‐Lincoln and sequenced on a NextSeq 2000 instrument at the National Jewish Health Genomics Facility using a P3 100c kit. Raw data were processed using Cell Ranger and subsequent analyses were performed using Seurat v.5.3.0. In brief, FASTQ files containing sequenced reads were mapped to the mouse reference genome (GRCm39). After Seurat objects were generated, low‐quality cells were removed in the merged Seurat object after quality control filtering (500 < nCount_RNA < 30 000, 200 < nFeature_RNA < 5000, reads mapped to hemoglobin genes < 3%, and mitochondrial reads < 10%); and the scDblFinder package (1.20.2) was used to detect doublets. After gene expression counts were normalized and scaled, the Principal Component Analysis was performed to reduce data dimensionality, and the batch effects were corrected using Harmony (1.2.3). The Harmony‐corrected data were analyzed for graph‐based clustering using the top 20 principal components at a resolution of 0.2. Cell clusters were manually annotated based on marker genes of each cell type from published single‐cell transcriptome studies [34, 35, 36]. To identify differentially expressed genes between the two phenotypes, we used the FindMarkers function in Seurat with the MAST test.

### 2.10 Oil Red O Staining and Quantification

The aortas were dissected from mice and fixed in 10% neutral buffered formalin for more than 1 day. After complete removal of perivascular adipose tissue, Oil Red O staining was performed. Briefly, samples were pre‐treated with propylene glycol, followed by incubation with Oil Red O solution (Electron Microscopy Sciences, cat# 26079‐15) for 2–3 h. Samples were then rinsed with 85% propylene glycol for 5 min, and subsequently washed repeatedly with PBS until the background was clear. Atherosclerotic lesion areas were quantified by en‐face analysis using Fiji/Image J. Briefly, RGB images were color‐deconvoluted into three channels using “FastRed FastBlue DAB” vector. The Colour_1 channel (red) was used to quantify Oil Red O‐positive areas, while the Colour_2 channel was used to quantify total aortic areas. For regional analysis, the aorta was segmented into three distinct regions of interest: the aortic arch, descending thoracic aorta, and abdominal aorta. Both the Oil Red O‐positive area and the total aortic area were quantified within each ROI for every aorta using the Analyze Particles function after the threshold was adjusted. Finally, the lesion percentage was calculated for each ROI using the following formula: (Oil Red O‐positive area/aortic area) × 100.

Aortic roots were embedded in O.C.T. (Optimal Cutting Temperature) compound for frozen sectioning. The embedded aortic roots were cut into serial cryostat sections at 6 μm. Frozen sections were fixed for 2–5 min in 10% formalin and stained in the preheated Oil Red O stain solution at 60°C for 6 min. Oil Red O positive areas were quantified using ImageJ.

### 2.11 Oil Red O Staining in the Liver and Quantification

Frozen liver sections (8 μm thickness) were prepared using FS800 cryostat (RWD) and used for histological detection of neutral lipid accumulation by Oil Red O staining. Propylene glycol (Fisher Scientific, Cat. No. P355‐4), Oil Red O stain (Electron Microscopy Sciences [EMS], cat# 26079‐15), Mayer's hematoxylin (Sigma‐Aldrich, cat# MHS15‐500 mL), and 10% formalin (Sigma‐Aldrich, cat# HT501128) were used for staining. An 85% propylene glycol solution prepared in distilled water was used for differentiation.

Briefly, frozen liver sections were equilibrated to room temperature for 30 min before staining. A 0.7% Oil Red O working solution was preheated to approximately 60 using a water bath. Sections were fixed in 10% formalin for 2–5 min and subsequently rinsed with tap water. Sections were then incubated in propylene glycol for 2 min, followed by incubation in the preheated 0.7% Oil Red O solution for 6 min. Excess stain was removed by differentiation in 85% propylene glycol for 1 min. Sections were rinsed twice with distilled water and counterstained with Mayer's hematoxylin for 1 min. Following two washes with tap water and two additional washes with distilled water, sections were mounted with an aqueous mounting medium and examined by light microscopy.

Oil Red O‐positive neutral lipid deposits were visualized as red to red‐orange staining, while nuclei appeared blue following hematoxylin counterstaining. Hepatic lipid accumulation was quantified by measuring the Oil Red O‐positive area in liver sections as described above for Oil Red O‐staining of aortas using ImageJ/Fiji software.

### 2.12 Hematoxylin and Eosin (H&E) Staining and Quantification

Frozen sections of aortic roots were stained with Hematoxylin and eosin (H&E) at the Nebraska Veterinary Diagnostic Center of the University of Nebraska‐Lincoln following the standard laboratory protocols. Briefly, frozen sections were fixed in methanol for 2 min and processed through 95% alcohol for 1 min and water for 1 min. Sections were then stained in hematoxylin for 2 min, rinsed in water for 2 min, differentiated in an acid rinse for 45 s, and washed in water for 2 min. After bluing for 2 min and a 2‐min water rinse, the sections were dehydrated in 95% alcohol for 1 min and counterstained with eosin for 30 s. Finally, the tissues were dehydrated through three stations of 100% alcohol (4 min total), cleared in three stations of Formula 83 xylene substitute (3 min total), and coverslipped using a mounting medium. Immune cell infiltration within atherosclerotic lesions was quantified using Fiji/ImageJ. Briefly, the atherosclerotic plaque area was defined as the region of interest (ROI) in RGB images. Individual ROIs were saved to the ROI Manager and merged into a single composite selection. This composite ROI was then color‐deconvoluted using the “H&E” vector to separate the image into three channels. A threshold was applied to the Colour_1 (Hematoxylin/Nuclei) channel to segment the dark blue mononuclear cell nuclei. Overlapping nuclei were separated using the watershed function (Process > Binary > Watershed), and the Analyze Particles tool was applied to quantify mononuclear cell nuclei. The Size parameter was set to 5–characterizing pixels (5‐infinity), and the Circularity was filtered between 0.5 and 1.00. The absolute cell count was normalized to the total lesion area to evaluate the fold change between the fl/fl and iECKO conditions.

### 2.13 Immunofluorescence Staining and Quantification

Liver and aortic roots were embedded in OCT Tissue Tek (Fisher Scientific, cat# 4585) and cut into serial cryostat sections at 8 μm (liver) or 6 μm (aortic roots) using FS800 cryostat (RWD). Tissue sections were allowed to equilibrate to room temperature before staining and were processed using standard immunofluorescence procedures. Briefly, tissue sections were fixed with cold acetone/methanol (1:1 volume) at 4°C for 10 min, permeabilized and blocked with 1% BSA in 0.3% Triton‐X‐100 1× dPBS for 1 h at RT, followed by primary antibodies incubation in blocking buffer (1% BSA and 0.3% Triton‐X‐100 in 1× dPBS) at 4°C for overnight and secondary antibodies in the blocking buffer for 1 h at RT with wash between steps. Primary antibodies used for staining include anti‐p21 (Abcam, cat# ab188224; 1:100 dilution), anti‐CD31 (BD Pharmingen, cat# 550274; 1:100 dilution), anti‐CD68 (BioLegend, cat# 137001; 1:500 dilution), anti‐CD163 (Abcam, cat# ab182422; 1:200 dilution), anti‐Ki67 (Cell Signaling Technology, cat# 9129S; 1:400 dilution), anti‐Matrin‐3 (FORTIS Life Sciences, cat# A300‐591A; 1:500 dilution) were used for staining. Secondary antibodies were used at 1:200 dilution, including Cy3‐Goat Anti‐Rat IgG (cat# 112‐165‐143) from the Jackson ImmunoResearch and DyLight 649 Goat Anti‐Rabbit IgG Antibody (H + L) (cat# DI‐1659) and DyLight 549 Horse Anti‐Mouse IgG (H + L) (cat# DI‐2549) from the Vector Laboratories. For nuclear visualization, stained sections were counterstained with Hoechst 33342 (Thermo Fisher Scientific, cat# 62249) at a 1:2000 dilution. Images were captured using Nikon Ti‐2 inverted fluorescence microscope or A1R‐Ti2 confocal system. Images were quantified using ImageJ/Fiji software.

### 2.14 Lipid Levels in the Plasma and Liver

Blood was collected from mice by cardiac puncture into 1.5 mL tubes pre‐treated with 0.5 M EDTA. The blood was gently mixed and kept on ice until centrifugation. All samples were centrifuged at 1500 × g for 15 min at 4°C. The plasma was then gently transferred into new 1.5 mL tubes, and 2 μL of plasma was used for each assay after appropriate dilution. Liver lipids were extracted according to the manufacturer's instructions (BioVision, cat# K216‐50) and used for subsequent assays at appropriate dilutions. Triglyceride and total cholesterol levels were measured using kits from Thermo Scientific (cat# TR22421 and cat# TR13421, respectively), and LDL cholesterol was measured using a kit from Crystal Chem (cat# 79980).

### 2.15 Plasma Levels of AST and ALT

Plasma AST and ALT activities were measured using colorimetric, NADH‐coupled kinetic assays (Cayman Chemical #701640 and #700260), with absorbance at 340 nm recorded at ~60‐s intervals over a 30‐min kinetic read. For each well, the initial linear reaction rate was determined empirically by fitting ordinary least‐squares regression to a series of candidate time windows anchored at the first timepoint and selecting the longest window achieving *R* ^2^ ≥ 0.95 (minimum four points, or three points for wells with an unusually rapid initial burst); manual adjustments to window start or end points were applied for wells with non‐representative early readings or a gradual transition to plateau, respectively, and confirmed by visual inspection against the raw kinetic trace. Enzyme activity (U/L) was calculated from the fitted initial rate using the assay kit's instructions. Each animal was assayed in technical triplicate per analyte, and per‐animal AST activity, ALT activity, and AST/ALT ratio were calculated as replicate means (no animal exceeded 20% CV). Three animals with AST, ALT, or AST/ALT ratio values elevated relative to their genotype group were confirmed as genuine biological outliers upon visual inspection of their kinetic traces and were excluded from statistical comparison.

### 2.16 RNA‐Pull Down Assay

Meg3 and its deletion mutants were subcloned into the pGEM‐3Zf(+) vector. The recombinant plasmids were linearized and used as templates for in vitro transcription to prepare biotinylated Meg3 and its antisense control RNA. The lncRNA pull‐down assay was performed as previously described [26] Briefly, HUVECs were collected and lysed to isolate nuclear fractions. Nuclear lysates were pre‐cleared and incubated with folded biotinylated sense or antisense Meg3 RNA. RNA‐protein complexes were captured with blocked streptavidin beads, followed by thorough washing. Bound proteins were eluted and analyzed by western blot.

### 2.17 RNA Immunoprecipitation Assay

RNA immunoprecipitation was performed as our previous study described [26]. Briefly, HUVECs were fixed, neutralized, and rinsed with PBS, followed by lysis on ice. Cell lysates were diluted, sonicated, and centrifuged to collect the supernatant. Cleared lysates were incubated overnight at 4°C with Dynabeads Protein G (Invitrogen, cat# 10004D) conjugated to control IgG (Bethyl Laboratories, cat# P120‐101) or anti‐Matrin‐3 antibody (Bethyl Laboratories, cat# A300‐591A). After sequential washing, bound RNA was eluted and extracted, then reverse‐transcribed. The enrichment of lncRNA Meg3 was determined by qPCR, and results were presented as the relative percentage of Meg3 in Matrin‐3 immunoprecipitants compared with the input.

### 2.18 Glucose and Insulin Tolerance Tests

Glucose tolerance tests (GTTs) and insulin tolerance tests (ITTs) were performed as previously reported [27]. Mice were fasted for 12 h for GTTs, then intraperitoneally injected with D‐glucose at 1.0 g/kg body weight. For ITTs, mice were fasted for 6 h and received intraperitoneal insulin (Humulin R) at 0.75 U/kg. Blood glucose was measured at baseline and 15, 30, 60, 90, and 120 min post‐injection using a One Touch Ultra Glucometer.

### 2.19 Western Blot Analysis

Cells were harvested by scraping and lysed in RIPA buffer supplemented with a protease inhibitor cocktail. The lysates were centrifuged at 12000 × g for 15 min, and the supernatants were collected. Protein concentrations were determined using the Pierce BCA Protein Assay Kit. To detect Matrin‐3 and other proteins, equal amounts of protein lysates were separated by SDS‐PAGE on a gradient gel. The following primary antibodies were used: anti‐Matrin‐3 (Bethyl Laboratories #A300‐591A; 1:1000 dilution), p53 (Santa Cruz Biotechnology, cat# sc‐126×; 1:5000 dilution), p21 (Cell Signaling Technology, cat# 2947S; 1:1000 dilution), p16 (Abcam, cat# ab108349; 1:1000 dilution), and anti‐GAPDH (Cell Signaling Technology, cat# 2118S; 1:10000 dilution). Protein bands were visualized using ECL Plus Western Blotting Detection Reagents (GE Healthcare, cat# RPN2236). Protein abundance was analyzed using ImageJ software.

### 2.20 Real‐Time Quantitative PCR Analysis

Total RNA from HUVECs was extracted according to the TRIzol manufacturer's instructions. cDNA was synthesized using the High‐Capacity cDNA Reverse Transcription Kit (Applied Biosystems, cat# 4368814). qPCR was performed on the CFX Connect Real‐Time System (Bio‐Rad) using 2× SYBR Green qPCR Master Mix (Selleck, cat# B21203). The primers of human genes used for qPCR were as follows: Matr3 forward 5′‐ATCAATGGAGCAAGTCACAGTC‐3′ and reverse 5′‐TGCAACATGAATGGATCACCC‐3′; Meg3 forward 5′‐GGGCATTAAGCCCTGACCTT‐3′ and reverse 5′‐CCTTGGGGAGGGAAACACTC‐3′; Gapdh forward: 5′‐ATGGGGAAGGTGAAGGTCG‐3′, reverse: 5′‐GGGGTCATTGATGGCAACAATA‐3′. The primers of mouse genes used for qPCR were as follows.

- *Col1a1*: 5′GCTCCTCTTAGGGGCCACT and 5′CCACGTCTCACCATTGGGG;
- *Timp1*: 5′CATGGAAAGCCTCTGTGGATATG and 5′GATGTGCAAATTTCCGTTCCTT;
- *Tgfb1*: 5′GTCACTGGAGTTGTACGGCA and 5′GGGCTGATCCCGTTGATTTC;
- *Mmp2*: 5′TTTGCTCGGGCCTTAAAAGTAT and 5′CCATCAAACGGGTATCCATCTC;
- *Acta2*: 5′GTCCCAGACATCAGGGAGTAA and 5′TCGGATACTTCAGCGTCAGGA;
- *Cd68*: 5′TCGCCTAGTCCAAGGTCCAA and 5′GATGGGTACCGTCACAACCTC;
- *Cd163*: 5′ATGGGTGGACACAGAATGGTT and 5′CAGGAGCGTTAGTGACAGCAG;
- *Gapdh*: 5′AGGTCGGTGTGAACGGATTTG and 5′TGTAGACCATGTAGTTGAGGTCA.

### 2.21 Public GEO Datasets Analysis

We obtained and analyzed three transcriptomic datasets from the NCBI Gene Expression Omnibus (GEO) database. The datasets contain gene expression in human carotid arteries with atheroma plaque or distant macroscopically intact tissue (GSE43292), with early or advanced atherosclerotic plaque (GSE28829), and aortic valve tissue from calcific aortic valve disease (CAVD) and normal control (non‐CAVD) (GSE235995). Normalized counts in CPM were plotted for GSE235995. The datasets GSE43292 and GSE28829 were analyzed using GEO2R after the groups were defined. The expression values of genes were copied into a CSV file. Because the expression values were log2 transformed, we performed an inverse log2 transformation in R to determine the original normalized intensity signal for each gene. Subsequently, the normalized intensity signal values were plotted using the ggviolin function from the ggpubr (0.6.3) package in R (4.4.3).

### 2.22 Statistical Analysis

Statistical analyses were performed in RStudio using R 4.4.3. and R packages. Normality was evaluated using the Shapiro–Wilk test, and variance homogeneity was assessed using Levene's test. Comparisons between two groups were performed using two‐tailed Student's *t*‐test for normally distributed data, and the Mann–Whitney *U* test for data not normally distributed. Data are presented as mean ± SEM unless otherwise indicated. Statistical significance was defined as *p* < 0.05.

## 3 Results

### 3.1 Matrin‐3 Knockdown Induces Cellular Senescence in HUVECs

Our previous studies revealed that long noncoding RNA—maternally expressed gene 3 (Meg3) prevents cellular senescence in endothelial cells. We also identified Matrin‐3 as a candidate protein binding partner of Meg3 [26]. To confirm the result, we first did an RNA pull‐down assay followed by western blot analysis of Matrin‐3. This analysis revealed that the region encompassing Meg3 nucleotides 441–1024 is critical for the interaction between Meg3 and Matrin‐3 (Figure S1A). To confirm the interaction of endogenous Meg3 and Matrin‐3, we performed RNA immunoprecipitation and found robust enrichment of Meg3 in the Matrin‐3‐interacting RNA fraction compared to IgG control (Figure S1B). Since we found that Meg3 is a critical regulator of cellular senescence in human endothelial cells and in the vascular endothelium in mice [26, 27, 37], we next examined the role of endothelial Matrin‐3 in cellular senescence. Several hallmarks of cellular senescence were examined, including cell proliferation, senescence‐associated β‐galactosidase (SA‐β‐gal) activity, and mitochondrial dysfunction. EdU (5‐ethynyl‐2′‐deoxyuridine) incorporation and imaging revealed that Matrin‐3 knockdown reduced the number of proliferating cells by 36.1% in Human Umbilical Vein Endothelial Cells (HUVECs) (Figure 1A). In addition, we found that Matrin‐3 knockdown increased the levels of SA‐β‐gal activity by 2.2‐fold (Figure 1B). Next, we asked whether Matrin‐3 knockdown increases levels of superoxide in HUVECs. Superoxide‐specific MitoSOX staining and flow cytometry revealed that HUVECs with Matrin‐3 knockdown showed a 1.4‐fold increase in superoxide levels (Figure 1C). In summary, these data revealed that Matrin‐3 knockdown inhibits cell proliferation, induces SA‐β‐gal activity, and increases mitochondria‐born superoxide, demonstrating that Matrin‐3 has a protective role against cellular senescence in HUVECs.

**FIGURE 1:**
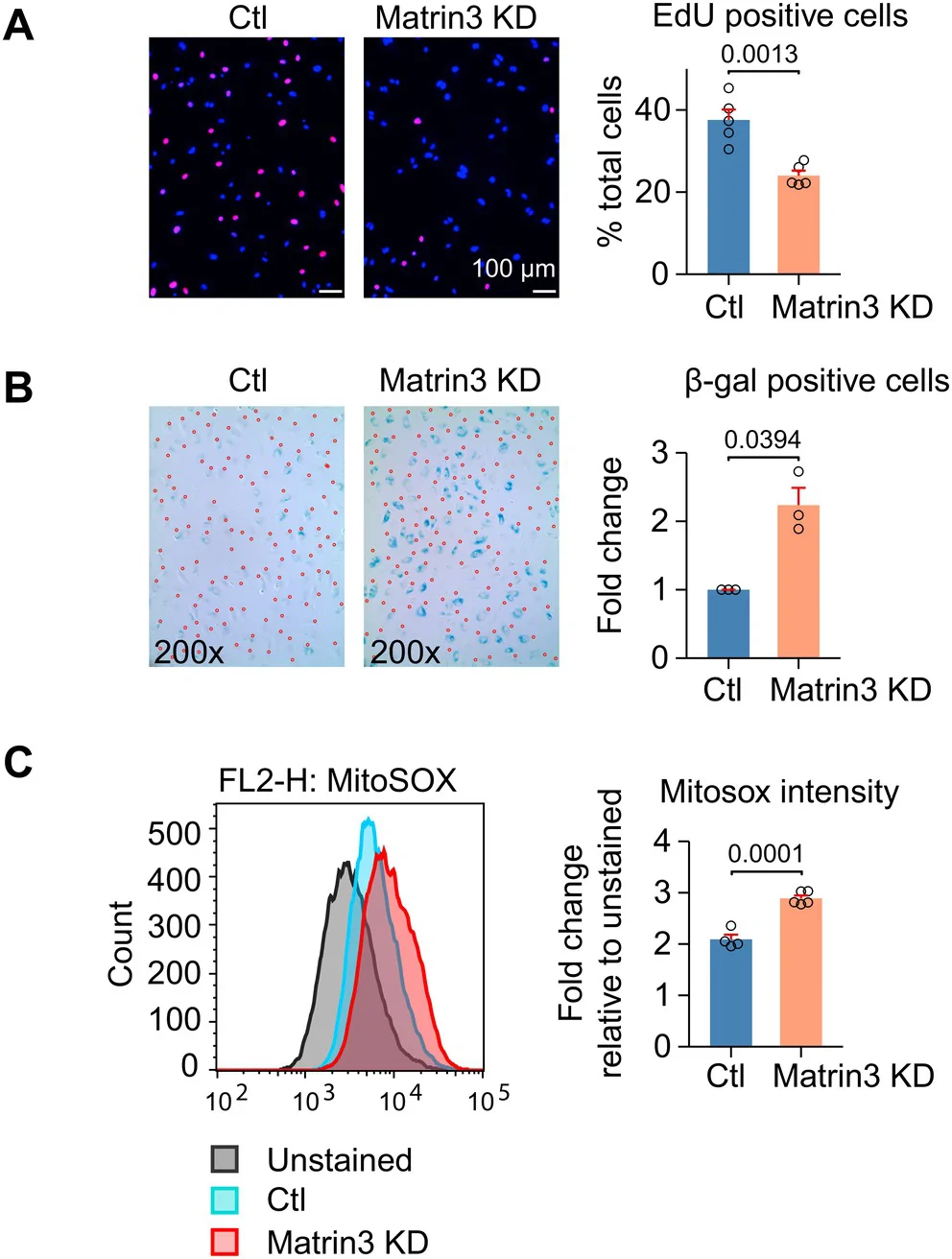
Matrin‐3 silencing induces cellular senescence in Human Umbilical Vein Endothelial Cells. (A) EdU incorporation assay in HUVECs with or without Matrin‐3 silencing. (B) SA‐β‐gal positive cells were calculated in HUVECs with or without Matrin‐3 silencing. (C) MitoSOX reagent staining followed by flow cytometry analysis in HUVECs with or without Matrin‐3 silencing. *n* = 4 and 5 for Ctl and Matrin‐3 KD respectively. Statistical analysis was performed using a two‐tailed Student's t‐test, assuming equal variance for (A, C), and unequal variance (Welch's t‐test) for (B). Data were pooled from three independent experiments; quantification was based on 32 images per condition in (A), and 35 images for control (Ctl) versus 36 images for Matrin‐3 knockdown (KD) in (B). We added red dots to the images using Adobe Photoshop to denote individual cells. Data are presented as mean ± SEM.

### 3.2 Matrin‐3 Knockdown Alters the Endothelial Proteome

We reasoned that Matrin‐3 controls the expression of many proteins that are important in limiting cellular senescence. Thus, we examined the effects of Matrin‐3 knockdown on the expression of proteins using TMT10‐plex labelling followed by mass spectrometry (Figure 2A). Principal component analysis (PCA) revealed that PC1 and PC2 account for 44.3% and 35.9%, respectively, of the variability within the proteomic data (Figure 2B). We found that biological replicates of each condition are clustered together. While the samples treated with two different Matrin‐3 shRNAs are separated, two clusters of them are further apart from the cluster of control shRNAs (Figure 2B), suggesting Matrin‐3 knockdown is the primary factor separating these samples. Indeed, we identified 536 differentially expressed proteins (|fold change| > 1.5 and *p*_adj_ < 0.05) (Figure 2C). Among them, 187 proteins were upregulated, and 349 proteins were downregulated. The gene symbols of differentially expressed proteins (*p*_adj_ < 0.05) were used for bioinformatics analysis using Enrichr [38]. Several Hallmark and KEGG pathways related to cell cycle, p53 signaling, DNA damage repair, cellular senescence, cell death, and lipid metabolism are enriched (Figure 2D). Consistent with the biological insights revealed by Enrichr, the protein expression of p53 and its target genes, such as SESN1, SESN2, VWCE, TP53I3 are induced by Matrin‐3 knockdown (Figure 2C), while the protein expression of Cyclin B1 (encoded by CCNB1), Cyclin B2 (CCNB2), DNA topoisomerase II‐alpha (TOP2A), and BUB1 Mitotic Checkpoint Serine/Threonine Kinase B (BUB1B) is reduced (Figure 2C). Western blot analysis revealed that silencing Matrin‐3 with two independent shRNAs successfully depleted its protein expression in HUVECs by 85.4% (shRNA1) and 76.7% (shRNA2) (Figure 2E). Importantly, Matrin‐3 deficiency upregulated the protein expression of p53 by 1.58‐fold and 2.38‐fold, p21 by 2.17‐fold and 7.05‐fold, and p16 by 1.99‐fold and 2.72‐fold for shRNA1 and shRNA2, respectively (Figure 2E). These proteins are important regulators of DNA replication and cell cycle progression. These data demonstrate that Matrin‐3 safeguards DNA replication, cell cycle progression, and DNA damage stress response such as p53 signaling to maintain cellular homeostasis and function in HUVECs.

**FIGURE 2:**
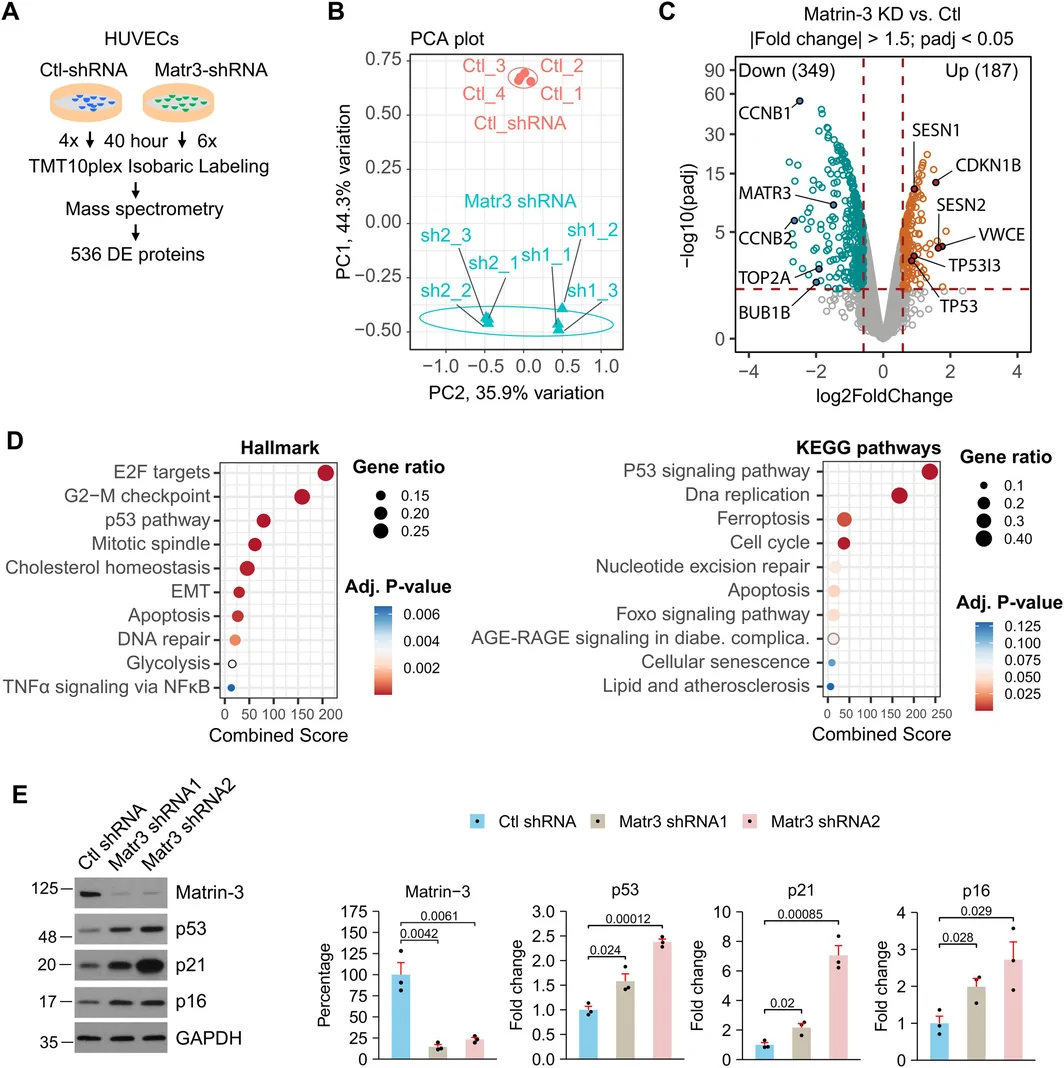
Matrin‐3 silencing alters the proteome in Human Umbilical Vein Endothelial Cells. (A) Schematic depicting the workflow of quantitative proteomics by TMT10 plex labeling followed by mass spectrometry and analysis. (B) Principal component analysis. (C) A volcano plot showing the differentially expressed proteins by quantitative proteomics in HUVECs. (D) Top Hallmark terms and KEGG pathways identified by Enrichr analysis. Gene symbols of the identified proteins were used for analysis. (E) Matrin‐3, p53, p21, and p16 protein expression by Western blot analysis.

### 3.3 Matrin‐3 Knockdown Alters the Endothelial Transcriptome

To examine whether Matrin‐3 regulates the same pathways in arterial endothelial cells as in HUVECs, we performed bulk RNA sequencing (RNA‐seq) to examine the effects of Matrin‐3 knockdown on the transcriptome in Human Coronary Artery Endothelial Cells (HCAEC) (Figure 3A). PCA showed clear separation between the control and Matrin‐3‐deficient samples along the first principal component (PC1), which captures most of the biological differences (Figure 3B). We identified 874 differentially expressed genes (|log2Fold change| > 0.585; *p*_adj_ < 0.05). Bioinformatic analysis of 3136 differentially expressed genes (*p*_adj_ < 0.05) using Enrichr [38] revealed that several Hallmark and KEGG pathways related to cell cycle, p53 signaling, DNA damage repair, cellular senescence, cell death, and lipid metabolism are enriched (Figure 3C), which corroborate the proteomic findings in HUVECs (Figure 2). We identified 116 (|log2Fold change| > 0.585; *p*_adj_ < 0.05) or 666 (|log2Fold change| > 0; *p*_adj_ < 0.05) genes that are differentially expressed at both mRNA levels in HCAECs and protein levels in HUVECs (Figure 3D). Enrichr analysis of the 666 overlapped genes revealed the same biological insights as Enrichr analysis of bulk RNA‐seq data or proteomic data, which substantiates the robustness of our approach, demonstrating that Matrin‐3 regulates DNA replication, p53 signaling, cell cycle, and cellular senescence in both venous and arterial endothelial cells.

**FIGURE 3:**
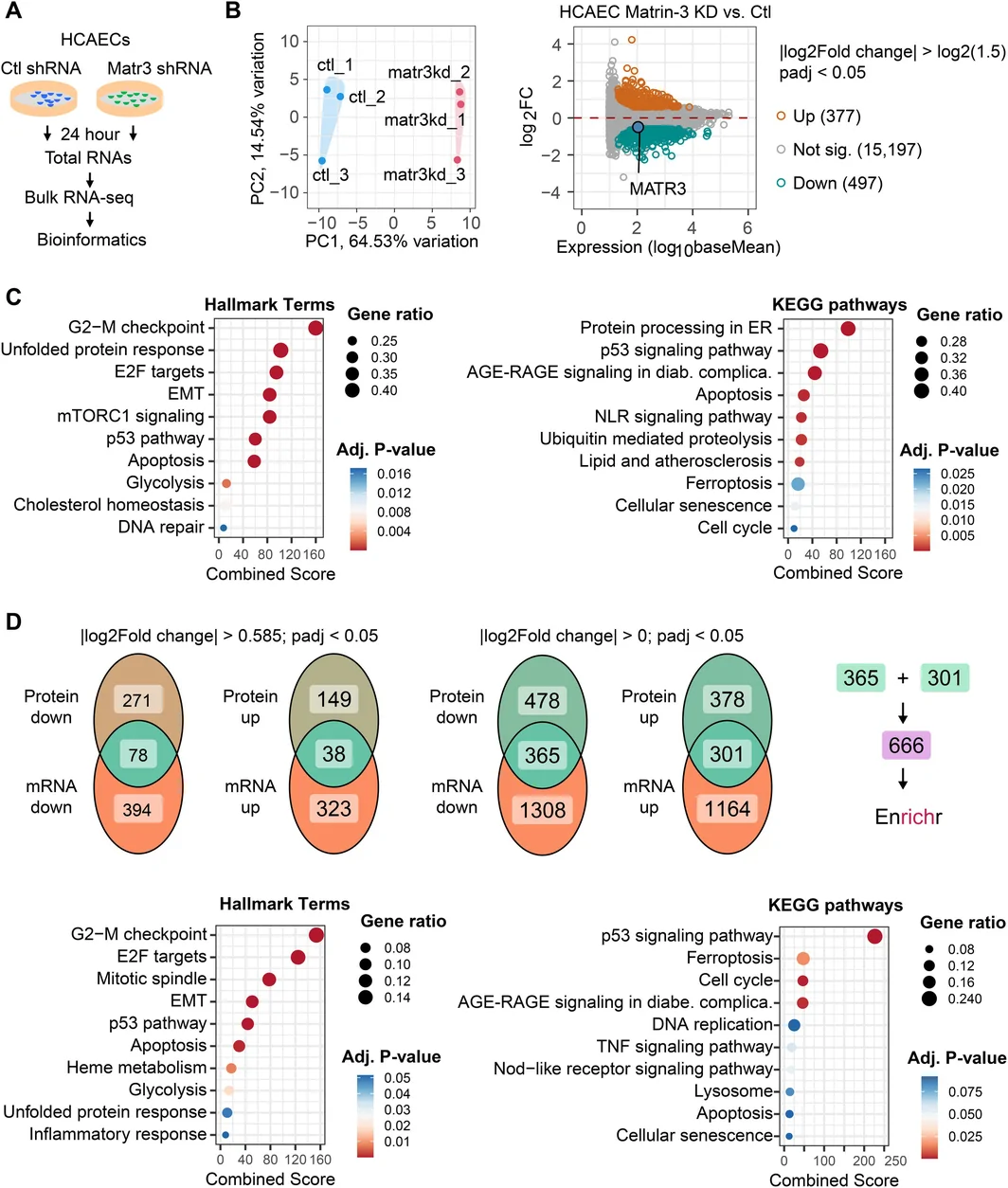
Matrin‐3 silencing alters the transcriptome in Human Coronary Artery Endothelial Cells. (A) Schematic depicting the workflow. (B) Principal component analysis showing the data variation among samples and a MA plot showing the differentially expressed genes identified by bulk RNA‐seq. (C) Top Hallmark terms and KEGG pathways identified by Enrichr analysis. 3136 differential genes (*p*_adj_ < 0.05) were used for analysis. (D) Gene symbols of differential proteins were overlapped with the differentially expressed genes identified by bulk RNA‐seq. Common differential genes were used for Enrichr analysis to identify enriched Hallmark terms and KEGG pathways.

Because Matrin‐3 is a protein binding partner of long non‐coding RNA Meg3, we examined the effects of Matrin‐3 silencing on Meg3 expression by qPCR; we found that 86.1% of Matrin‐3 reduction at mRNA levels is associated with a 45.3% reduction of Meg3 in HUVECs (Figure S1C), suggesting that Matrin‐3 is required for maintaining the levels of Meg3.

### 3.4 Endothelial Cell‐Specific Matrin‐3 Knockout Promotes Atherosclerotic Lesion Formation in Mice

Since we found that Matrin‐3 knockdown induces cellular senescence of endothelial cells, we asked whether Matrin‐3 deficiency promotes plaque formation in a mouse model of atherosclerosis. Following tamoxifen administration, Matr3 gene was efficiently deleted in the vascular endothelium, resulting in 35.5% and 53.0% Matrin‐3 protein reduction in the aortic root endothelial cells and hepatic endothelial cells, respectively (Figure S2). Matrin‐3 endothelial cell‐specific deficiency had no effects on body weight throughout the experiments (Figure S3A). Glucose tolerance and insulin tolerance tests revealed that it did not affect blood glucose levels (Figure S3B), suggesting that endothelial Matrin‐3 is not involved in regulating insulin signaling and glucose homeostasis. Oil Red O staining of the aortas revealed that endothelial Matrin‐3 deficiency significantly increased lesion formation, resulting in a 1.41‐fold increase in the whole aorta (Figure 4A), a 2.63‐fold increase in the descending thoracic aorta, and a 2.80‐fold increase in the abdominal aorta. In contrast, it did not significantly alter lesion formation in the aortic arch (1.30‐fold increase; *p* = 0.133) (Figure S4A). We also observed 42.3% increase in Oil Red O positive areas in the aortic sinus (Figure 4B). H&E staining revealed a mild and significant increase in the number of mononuclear cells in atherosclerotic lesions (Figure 4C; Figure S4B). However, we did not observe a difference in CD68‐ or CD163‐positive areas between groups (Figure S4C,D). Consistent with our observation that Matrin‐3 silencing causes endothelial senescence in vitro (Figure 1), endothelial Matrin‐3 deficiency induces the number of CD31 and p21 double positive cells at aortic roots (Figure 4D). These results demonstrate that endothelial cell‐specific Matrin‐3 deficiency promotes the formation of atherosclerotic plaques independent of macrophage infiltration in mice.

**FIGURE 4:**
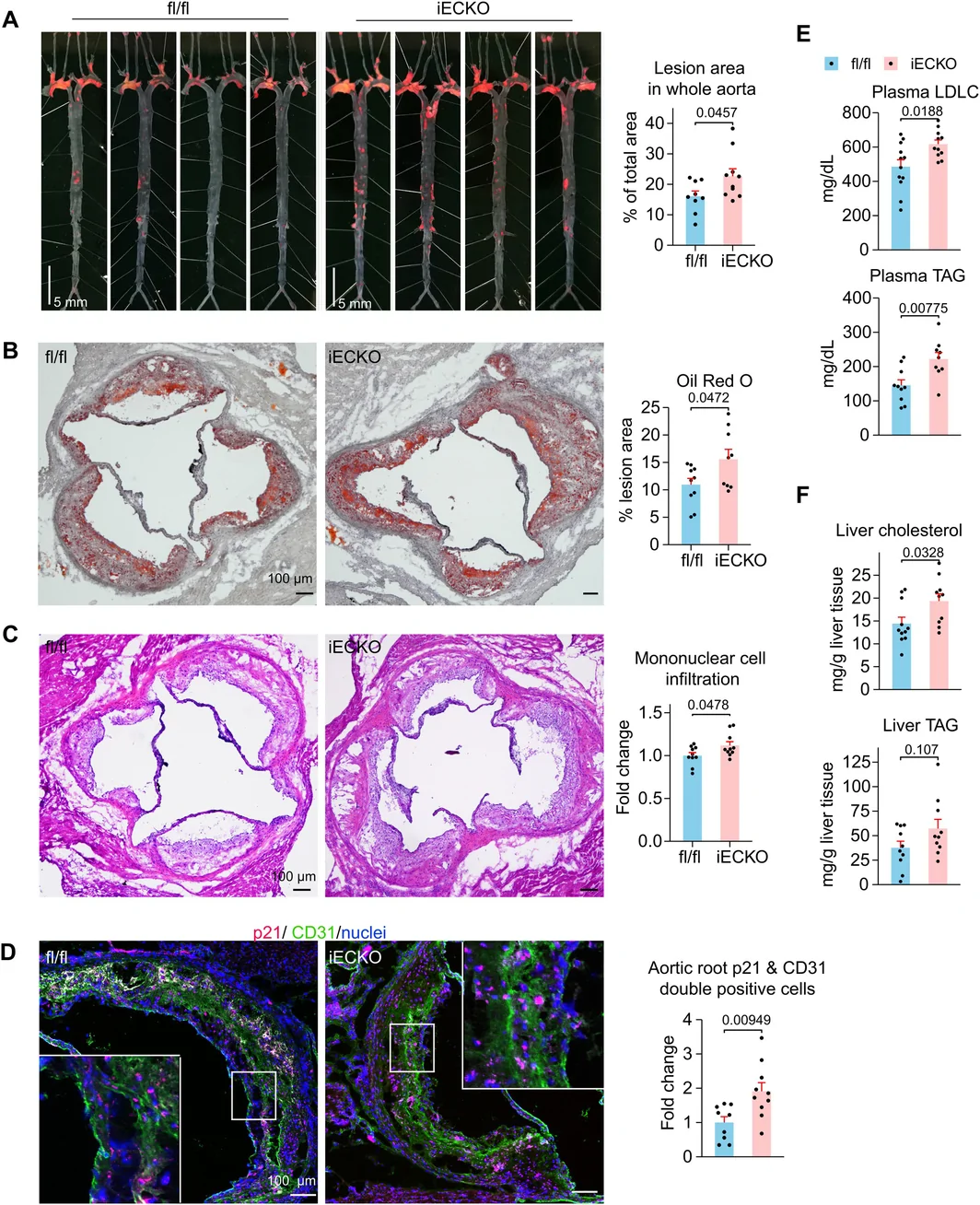
Endothelial cell‐specific Matrin‐3 deficiency accelerates the formation of atherosclerotic lesions. (A) Atherosclerotic lesion areas were revealed by Oil Red O staining. (B) Oil Red O staining on the aortic root sections. Images from 2 to 3 valves per mouse sample were used for quantification. The Oil Red O‐positive area was normalized by the total lesion area. (C) H&E staining and quantification of mononuclear cell infiltration in lesions. (D) CD31, p21, and nuclei (DAPI) immunostaining on the aortic root sections. Images from 2 to 3 valves per mouse sample were used for quantification. The number of CD31 and p21 double positive cells was counted. (E) Plasma levels of LDL cholesterol (LDLC) and triglycerides (TAG). (F) The levels of total cholesterol and triglycerides in the livers. Data represent mean ± SEM; *n* = 9–11 mice per group.

Hyperlipidemia drives the development of atherosclerosis. To examine the effects of endothelial Matrin‐3 deficiency on lipid homeostasis, we measured the plasma levels of low‐density lipoprotein (LDL) cholesterol and triglycerides and the levels of total cholesterol and triglycerides in the livers. The plasma levels of LDL cholesterol and triglycerides increased by 27.0% and 52.5%, respectively, in iECKO mice compared to fl/fl mice (Figure 4E), accompanied by elevated hepatic total cholesterol and triglycerides by 34.1% and 52.2%, respectively (Figure 4F). These data suggest that endothelial Matrin‐3 deficiency impairs lipid homeostasis in the circulation and liver, leading to hyperlipidemia.

### 3.5 Endothelial Cell‐Specific Matrin‐3 Deficiency Alters the Transcriptome in Aortic Intima

Because endothelial cell‐specific deficiency of Matrin‐3 increases the formation of atherosclerotic lesions, we next examined the effects of endothelial cell‐specific Matrin‐3 deficiency on the transcriptome in aortic intima. Aortic intima was peeled as previously described in our studies [32] for total RNA extraction and bulk RNA‐seq analysis (Figure S5A). We identified 696 differential genes (|log2FoldChange| > 0.585; *p* value < 0.05) (Figure S5A). Among them, 558 were increased and 138 were reduced. The upregulated genes include Maob, Plin1, Cidec, Pdk4, Bnip3, Il33, Slc1a5, Mtch2, Pparg, and Plin4, and the downregulated genes include Cd5l, Malat1, and Mki67. Bioinformatic analysis using Enrichr revealed that KEGG pathways such as oxidative phosphorylation, non‐alcoholic fatty liver disease, reactive oxygen species, PPAR signaling pathway are enriched (Figure S5B); and Hallmark terms such as oxidative phosphorylation, adipogenesis, reactive oxygen species pathway, and hypoxia are enriched (Figure S5C). In summary, endothelial cell‐specific Matrin‐3 deficiency alters the transcriptome in aortic intima, shifting the expression profile toward genes involved in endothelial inflammation, lipid uptake and storage, cell death, and metabolism reprogramming.

### 3.6 Endothelial Cell‐Specific Matrin‐3 Deficiency Reprograms the Transcriptome in Hepatocytes Associated With Genes Enriched for Lipid Metabolism and Inflammation

We found that endothelial Matrin‐3 deficiency resulted in increased levels of circulating and hepatic triglycerides and cholesterol. Liver is a key organ involved in lipid metabolism. We hypothesize that endothelial Matrin‐3 deficiency impairs hepatocyte function by altering the transcriptome in hepatocytes. To test this hypothesis, we performed single‐nucleus RNA‐seq (snRNA‐seq) analysis in the liver (Figure 5A). We analyzed 9070 Matrin‐3 fl/fl nuclei and 11 006 iECKO nuclei, respectively, and identified seven major cell types including hepatocytes (Hep), macrophages (Mac), hepatic stellate cells (HSC), EC, fibroblast/mesenchymal cells (Fib/mesen), leukocytes (Leuk), and cholangiocytes (Cholang) in the livers (Figure 5B,C). We found that the proportion of macrophages increased from 3.7% in the Matrin‐3 fl/fl livers to 5.7% in the iECKO livers, and the proportions of leukocytes also slightly increased (3.6% to 4.4%; Figure 5D). We identified 979 differentially expressed genes (DEGs) in the hepatocyte cluster (*p*_val_adj < 0.05 & |avg_log2FC| > 0.585; Figure 5E). Notably, the expression of several genes in hepatocytes was induced by endothelial Matrin‐3 deficiency, which promotes lipid droplet formation and accumulation (*Cidea* and *Fitm1*), the biosynthesis of triglycerides (*Scd1*), and the progression of metabolic dysfunction‐associated steatotic liver disease (*Osbpl3* and *Scd1*) (Figure 5F). In addition, the expression of genes associated with inflammation and acute phase response also increased, including (*Cxcl10*, *S100a1*, and *Saa1*) (Figure 5F). In contrast, the expression of *Insig2* (a gene that suppresses lipogenesis) and *Ppargc1a* (a gene that promotes lipid catabolism) decreased (Figure 5F). The *Hmgcr* gene encodes HMG‐CoA reductase, the rate‐limiting enzyme in the endogenous synthesis of cholesterol. Its expression was likely reduced due to the feedback inhibition by the increased levels of liver cholesterol (Figure 4F). The expression of the target genes of constitutive androstane receptor (CAR), including *Cyp2b10*, *Cyp39a1*, *Ddit4*, and *Fmo2* is among the reduced genes in the iECKO hepatocyte cluster, suggesting that CAR signaling is impaired (Figure 5F). Consistent with this observation, Enricher analysis of all DEGs (*p*_val_adj < 0.05; 5975 genes) revealed that the transcriptomic changes in the hepatocyte cluster are overrepresented by the downregulated genes in *Rora* or *Ppara* knockout cells (Figure 5G). Retinoid‐related orphan receptor alpha (RORα encoded by *Rora*) is important in mediating the interaction between CAR and other co‐regulators to form active chromatin complex and turn on gene expression involved in xenobiotic and lipid metabolism [39]. Consistent with the results, Enrichr analysis revealed that Hallmark terms associated with xenobiotic metabolism, lipid metabolism, and inflammation were enriched in the Matrin‐3 iECKO hepatocytes (Figure 5H). These data suggest that endothelial Matrin‐3 deficiency reprograms the transcriptome in hepatocytes, which likely contributes to the increased lipid accumulation and inflammation in the liver through nuclear receptor signaling, indirectly promoting the formation of atherosclerotic lesions in the aorta through the increased circulating LDL cholesterol and triglycerides.

**FIGURE 5:**
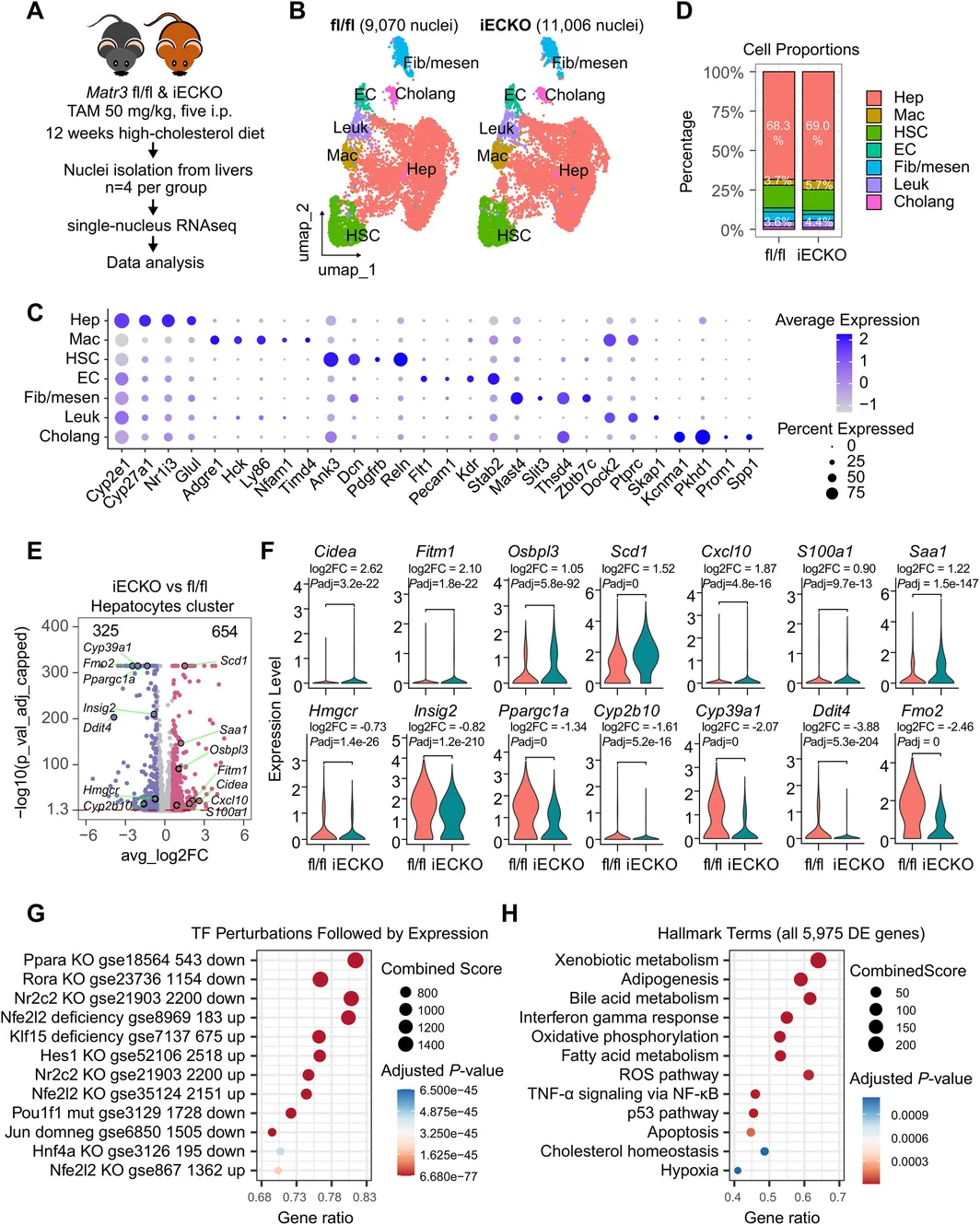
Endothelial cell‐specific Matrin‐3 deficiency alters the transcriptome in the liver. (A) Schematic depicting the workflow of single‐nuclei RNA‐seq (snRNA‐seq) analysis. Nuclei were isolated from eight livers (*n* = 4 for the fl/fl and iECKO samples, respectively) of mice fed on a high cholesterol diet for 12 weeks. (B) Uniform manifold approximation and projection (UMAP) of snRNA‐seq for the fl/fl (*n* = 9070 nuclei) and iECKO (*n* = 11 006 nuclei) samples after quantity control and data filtering using Harmony integration. (C) A dot plot depicts the expression of the marker genes for each cell type. (D) Cell proportion of annotated cell clusters in the fl/fl and iECKO samples. (E) The differentially expressed genes (DEGs; |avg_log2FC| > 0.585 and p_val_adj < 0.05) identified by the FindMarkers function in the Seurat package using the MAST test. (F) Violin plots show the expression of the selected genes in E. (G) TF perturbation followed by expression revealed by Enrichr analysis of the 5975 differentially expressed genes (p_val_adj < 0.05) in hepatocytes. (H) Enriched Hallmark terms were identified using Enrichr analysis of the 5975 differentially expressed genes (p_val_adj < 0.05) in the hepatocyte cluster between Matrin‐3 fl/fl and iECKO samples.

### 3.7 Endothelial Cell‐Specific Matrin‐3 Deficiency Causes Steatosis, Fibrosis, Inflammation, and Endothelial Senescence in the Liver

We further examined the effects of endothelial Matrin‐3 deficiency on steatosis, fibrosis, inflammation, and endothelial senescence in liver. Endothelial Matrin‐3 deletion caused a 1.96‐fold increase in the Oil Red O‐positive area in frozen liver sections (Figure 6A), while it did not affect lipid droplet size (1.52‐fold increase; *p* = 0.133). Endothelial Matrin‐3 deletion also resulted in a 1.97‐, 2.40‐, and 1.41‐fold increase in *Col1a1*, *Timp1*, and *Cd68* expression, respectively, in the livers (Figure 6B). However, it did not alter the expression of *Tgfb1* (1.35‐fold increase; *p* = 0.0684), *Mmp2* (1.29‐fold increase; *p* = 0.263), *Acta2*, and *Cd163* (29% reduction, *p* = 0.605). In addition, endothelial Matrin‐3 deletion increased the number of p21 and CD31 double positive cells by 2.18‐fold in the liver (Figure 6C). Moreover, endothelial Matrin‐3 deletion tended to increase plasma levels of ALT and AST (1.58‐fold and 1.18‐fold increase, respectively), though this trend did not reach statistical significance (Figure 6D). These changes are associated with 53.0% Matrin‐3 protein reduction in the hepatic endothelial cells (Figure S2B). These data demonstrate that endothelial cell‐specific Matrin‐3 deficiency causes steatosis, fibrosis, inflammation, and endothelial senescence in the liver in the absence of severe liver damage.

**FIGURE 6:**
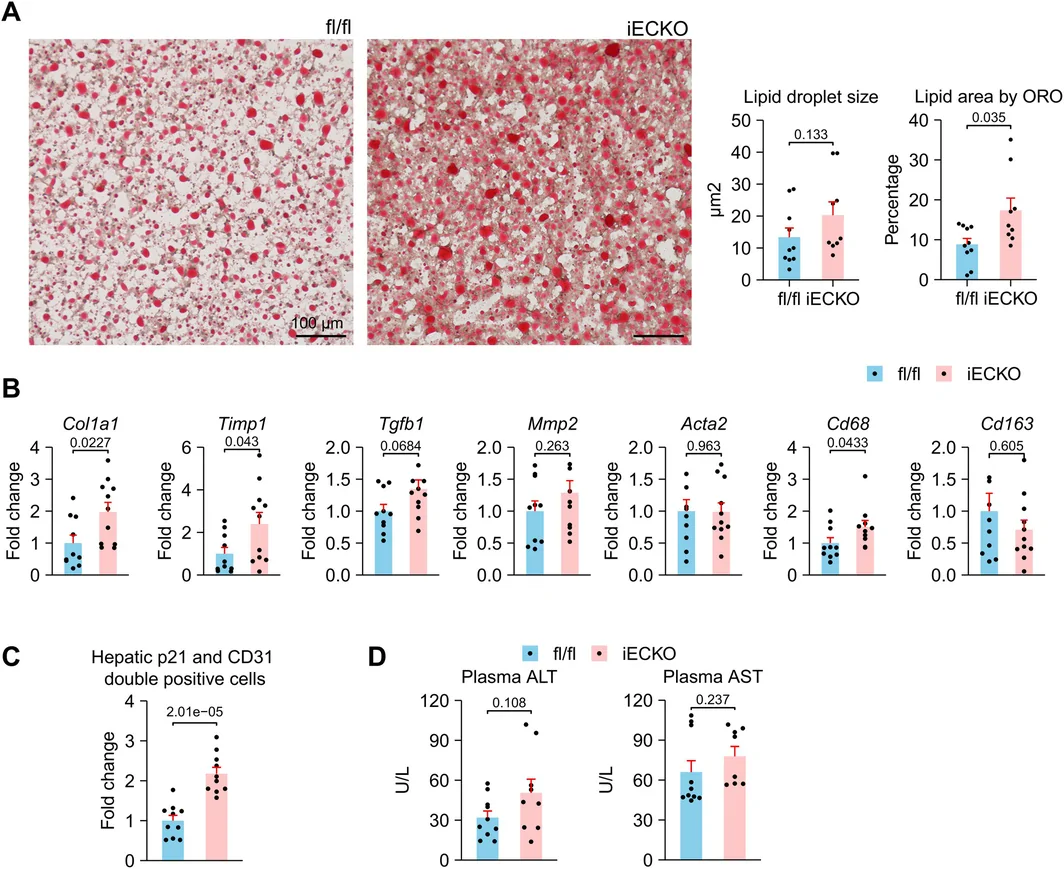
Endothelial cell‐specific Matrin‐3 deficiency causes steatosis, fibrosis, inflammation, and endothelial senescence in the liver. (A) Oil Red O staining of frozen liver sections and quantification. (B) qPCR analysis of the indicated genes in the livers of fl/fl and iECKO mice. (C) Quantification of dual immunostaining of p21 and CD31 in the livers. (D) Plasma levels of ALT and AST. Data represent mean ± SEM; *n* = 9–11 mice per group.

### 3.8 Matrin‐3 Expression Is Reduced in Human and Mouse Atherosclerotic Arteries and Aortic Valves

To examine whether Matrin‐3 expression is altered in the vessel wall during atherosclerosis, we first examined Matrin‐3 mRNA expression in human arteries. We obtained and analyzed three datasets from the NCBI Gene Expression Omnibus (GEO) database. The relative expression of Matrin‐3 mRNA was significantly reduced in the atheroma plaques, advanced atherosclerotic plaques, and aortic valve tissue from calcific aortic valve disease than in their control tissues, respectively (Figure 7A). We also found that it was reduced by 15.3% in thoracic and abdominal aortas and 31.4% in the aortic arches of atherosclerotic mice (Figure 7B). These results revealed that Matrin‐3 expression is reduced in human and mouse atherosclerotic arteries, indicating its potential role in atherosclerosis in humans.

**FIGURE 7:**
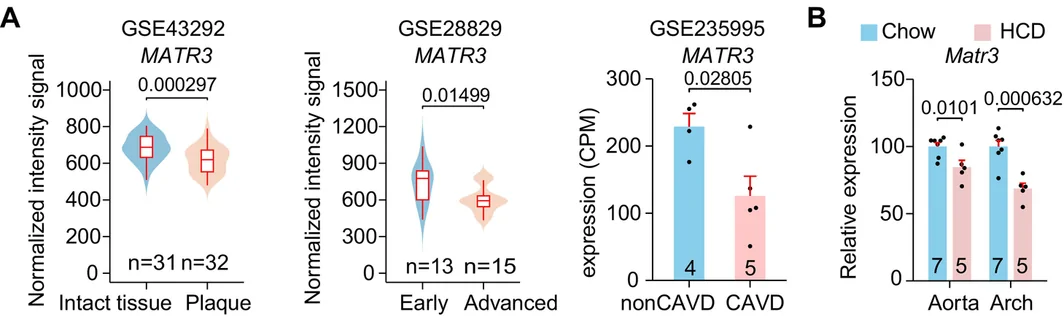
Matrin‐3 expression is reduced in human and mouse arteries under pathological conditions. (A) Matrin‐3 mRNA expression in human carotid arteries or aortic valve tissues. (B) Matrin‐3 mRNA relative expression in mouse aortas or aortic arches. Data show mean ± SEM for Matr3 expression in GSE235995 and (B). In the boxplots, the bottom edges represent the first quartile (25th percentile), the top edges represent the third quartile (75th percentile), and the middle lines represent the median.

## 4 Discussion

In this study, we identify endothelial Matrin‐3 as an important regulator of cellular senescence and atherosclerosis. Matrin‐3 regulates the expression of hundreds of genes and proteins involved in DNA damage responses, cell‐cycle control, p53 signaling, metabolism, and cellular senescence. Matrin‐3 expression is reduced in atherosclerotic lesions in mice and humans, whereas endothelial cell‐specific Matrin‐3 deficiency accelerates atherosclerotic lesion formation in mice. Importantly, endothelial Matrin‐3 deficiency is associated with both local transcriptional alterations in the aortic wall and systemic metabolic abnormalities characterized by hepatic lipid accumulation and increased circulating LDL cholesterol (LDLC) and triglycerides (TAG). Together, our findings demonstrate that endothelial Matrin‐3 deficiency is associated with coordinated alterations in endothelial homeostasis, local vascular responses, and systemic metabolism in the setting of accelerated atherosclerosis.

Matrin‐3 was identified as a protein‐binding partner of the long noncoding RNA Meg3 (Figure S1). We also found that Matrin‐3 silencing reduces Meg3 expression in human endothelial cells (Figure S1). We previously demonstrated that Meg3 limits DNA damage and cellular senescence in endothelial cells [26, 27, 37]. These observations suggest that the interaction between Meg3 and Matrin‐3 may represent an important mechanism for maintaining endothelial homeostasis and function under stress conditions. To define the molecular programs regulated by Matrin‐3, we integrated proteomic analysis of HUVECs with transcriptomic analysis of HCAECs. Cross‐referencing these datasets identified 666 differentially expressed proteins whose corresponding mRNAs were also altered (Figure 3D). Despite differences in endothelial cell origin and experimental platforms, the substantial overlap between the transcriptomic and proteomic responses strengthens the evidence that Matrin‐3 regulates conserved molecular programs involved in endothelial stress responses and cellular senescence.

Our studies further reveal an important role for endothelial Matrin‐3 in limiting atherosclerosis. Matrin‐3 regulates numerous proteins involved in DNA damage responses, cell‐cycle progression, p53 signaling, and cellular senescence. Consistent with these in vitro findings, RNA‐seq analysis of the aortic intima identified 696 differentially expressed genes between Matrin‐3 fl/fl and iECKO mice (Figure S5A), demonstrating substantial transcriptional remodeling within the vascular compartment following endothelial Matrin‐3 deletion. These genes are involved in pathways relevant to vascular inflammation, cellular metabolism, lipid handling, mitochondrial function, cell survival, and proliferation. For example, *Maob* encodes monoamine oxidase B, which can contribute to vascular oxidative stress and inflammation [40, 41], whereas *Pdk4* functions as a metabolic switch that regulates endothelial substrate utilization [42]. *Bnip3* and *Mtch2* regulate mitochondrial function, metabolism, and cell survival [43, 44], and *Il33* has been implicated in inflammatory, angiogenic, and thrombotic endothelial responses [45, 46]. *Slc1a5* has also been linked to paracrine signaling by senescent endothelial cells [47].

The aortic transcriptomic changes additionally included genes involved in lipid handling and atherosclerosis. *Plin1*, *Cidec*, and *Plin4* regulate cellular lipid storage [48, 49], whereas *Pparg* is a major transcriptional regulator of lipid metabolism and has complex cell type‐dependent functions in atherosclerosis [50, 51]. *Cd5l* encodes a secreted protein implicated in inflammatory responses and foam‐cell formation [52]. Changes in *Malat1*, which contributes to endothelial survival and vascular growth [53], and *Mki67*, a marker of cellular proliferation [54], further suggest broad remodeling of cellular responses within the aortic intima. Collectively, these findings indicate that endothelial Matrin‐3 deficiency is associated with local vascular alterations involving inflammation, lipid handling, mitochondrial function, cell death, and metabolic reprogramming. Thus, our data do not exclude—and instead support—a contribution of local vascular mechanisms to the enhanced atherosclerosis observed in Matrin‐3 iECKO mice.

The protective actions of Matrin‐3 within the vessel wall may extend beyond endothelial cells. Matrin‐3 has been reported to alleviate inflammatory responses during atherosclerosis by promoting mRNA m6A modification and m6A‐mediated mRNA decay in macrophages [29]. Together with our findings, these observations suggest that Matrin‐3 can exert atheroprotective functions in multiple vascular cell types through distinct molecular mechanisms. In the present study, however, Matrin‐3 deletion was restricted to endothelial cells, allowing us to specifically demonstrate the importance of endothelial Matrin‐3 in protecting against atherosclerotic lesion formation.

An important finding of the present study is that the consequences of endothelial Matrin‐3 deficiency extend beyond the vascular wall. Endothelial cell‐specific Matrin‐3 deletion increased plasma LDLC and TAG and increased hepatic total cholesterol and triglyceride levels. These metabolic abnormalities were accompanied by hepatic steatosis, fibrosis, inflammation, and endothelial senescence. Importantly, single‐nucleus RNA‐seq (snRNA‐seq) of the liver provided cell‐resolved evidence that an endothelial‐specific genetic perturbation is associated with substantial transcriptional reprogramming in other hepatic cell populations, particularly hepatocytes. Differentially expressed genes in hepatocytes were enriched in pathways related to lipid metabolism and inflammation (Figure 5), consistent with a functional link between endothelial Matrin‐3 deficiency and hepatocyte transcriptional responses.

Liver endothelial cells are positioned to regulate hepatic homeostasis through communication with hepatocytes and other resident cell types. Previous studies have demonstrated that liver endothelial cells can regulate hepatocyte function through multiple signaling mechanisms [55, 56, 57, 58, 59]. Thus, our observation that endothelial‐specific Matrin‐3 deletion is accompanied by hepatocyte transcriptional reprogramming, hepatic lipid accumulation, and altered circulating lipids is consistent with an endothelial‐to‐hepatocyte signaling mechanism. Importantly, however, the present experiments do not establish that endothelial senescence alone is sufficient to cause hepatocyte dysfunction or define the specific endothelial‐derived mediator(s) responsible for these effects. Rather, our findings identify a previously underexplored relationship between endothelial Matrin‐3, endothelial senescence, and hepatic metabolic homeostasis and provide a basis for future studies to determine whether, and through which molecular signals, endothelial Matrin‐3 deficiency influences hepatocyte function.

One potential pathway linking endothelial Matrin‐3 deficiency to hepatocyte dysfunction is altered constitutive androstane receptor (CAR) signaling. We previously demonstrated that Matrin‐3 is required for CAR signaling and function in hepatocytes under metabolic stress by stabilizing CAR mRNA [28]. CAR acts as an anti‐lipogenic factor under metabolic stress conditions. CAR can induce *Insig‐1*, which inhibits SREBP‐1 maturation and lipogenesis [60], and can promote hepatic cholesterol disposal by increasing expression of *Cyp7a1*, the rate‐limiting enzyme in bile acid synthesis, as well as transporters involved in bile acid excretion [61, 62]. Consistent with a potential role for this pathway, CAR activation has been reported to reduce very‐low‐density lipoprotein production and hepatic triglyceride content and to repress several genes involved in lipogenesis [63]. CAR signaling is also sensitive to inflammatory stimuli, including interleukin‐1β, lipopolysaccharide, and infection [64, 65].

Notably, our liver snRNA‐seq analysis revealed reduced expression of several CAR target genes specifically within the hepatocyte cluster (Figure 5), consistent with impaired CAR signaling in hepatocytes of Matrin‐3 iECKO mice. Because Matrin‐3 is deleted in endothelial cells rather than hepatocytes in this model, these findings raise the possibility that endothelial dysfunction and/or the associated inflammatory and metabolic environment indirectly suppresses hepatocyte CAR signaling. Such impairment could contribute to increased hepatic lipid accumulation and elevated circulating cholesterol and triglycerides. However, the present data do not establish a direct causal pathway from endothelial Matrin‐3 deficiency to CAR suppression, and the molecular mediators connecting these events remain to be identified. Future studies using targeted genetic and molecular approaches will be required to determine whether restoration of hepatocyte CAR signaling can rescue the hepatic metabolic phenotype observed following endothelial Matrin‐3 deficiency.

Our findings therefore demonstrate that endothelial Matrin‐3 deficiency is associated with both local vascular alterations and systemic metabolic dysfunction in the setting of accelerated atherosclerosis. The aortic intimal RNA‐seq data demonstrate substantial transcriptional changes within the vascular compartment, whereas liver snRNA‐seq reveals cell type‐specific transcriptional reprogramming of hepatocytes accompanied by hepatic steatosis, inflammation, fibrosis, and systemic lipid abnormalities. These findings are not mutually exclusive and raise the possibility that both local and systemic processes contribute to the enhanced atherosclerotic phenotype. Local endothelial dysfunction and senescence may alter the vascular microenvironment, whereas endothelial dysfunction in the liver may perturb hepatic lipid homeostasis and thereby influence the circulating lipid environment. However, the relative causal contributions of these local vascular and systemic metabolic alterations to atherosclerosis remain to be established.

Several questions remain to be addressed. Although liver snRNA‐seq allowed us to resolve cell type‐specific transcriptional responses to endothelial Matrin‐3 deficiency, the aortic RNA‐seq analysis was performed on the intimal compartment and therefore does not resolve the responses of individual vascular cell populations. Single‐cell or single‐nucleus transcriptomic analysis of the aorta could further define how endothelial Matrin‐3 deficiency alters endothelial, immune, smooth muscle, and other vascular cell populations and could identify additional intercellular signaling pathways. However, transcriptomic profiling alone would not establish the relative causal contributions of local vascular alterations versus hepatic lipid dysregulation to the enhanced atherosclerotic phenotype, nor would it determine the extent to which these phenotypes are specifically mediated by cellular senescence. Addressing these questions will require targeted mechanistic approaches, including cell type‐specific genetic manipulation, rescue experiments, and interrogation of candidate endothelial‐derived signals.

In summary, our study identifies Matrin‐3 as an important regulator of endothelial homeostasis, cellular senescence, and atherosclerosis. Matrin‐3 suppresses molecular programs associated with DNA damage and cellular senescence in human endothelial cells, and endothelial‐specific Matrin‐3 deficiency accelerates atherosclerosis in mice. Our findings further demonstrate that endothelial Matrin‐3 deficiency is associated with both extensive transcriptional remodeling in the aortic intima and cell type‐specific transcriptional alterations in the liver, including dysregulation of hepatocyte pathways involved in lipid metabolism, inflammation, and CAR signaling. These changes are accompanied by hepatic steatosis, fibrosis, inflammation, and increased circulating lipids. Together, these observations demonstrate that endothelial Matrin‐3 deficiency is associated with both local vascular and systemic metabolic alterations in the setting of accelerated atherosclerosis, although the relative causal contributions of these processes remain to be determined. Reduced Matrin‐3 expression in diseased human arteries and aortic valves further supports the potential relevance of this pathway to human vascular disease. Future studies defining how endothelial Matrin‐3 influences intercellular signaling and systemic metabolic homeostasis may reveal new opportunities for therapeutic intervention in cardiovascular and metabolic disease.

## Author Contributions

X.C. and X.S. conceived and designed the study, prepared the figures and drafted the manuscript. X.C., F.Y.T., R.N.Z., E.A., and E.C. conducted the experiments. Data analysis and interpretation was performed by X.C., X.S., F.Y.T., R.N.Z., and E.A. All authors contributed to the revision of the manuscript and approved the final version.

## Funding

This work was supported by the National Institutes of Health R01 grants HL150536, HL175569, DK145704 (to X.S.), NIGMS T32 2T32GM136593‐06 (R.N.Z.), and NIGMS (P30GM154608).

## Conflicts of Interest

The authors declare no conflicts of interest.

## Supporting information


**Figure S1:** Matrin‐3 is a protein binding partner of long noncoding RNA Meg3.
**Figure S2:** Matrin‐3 expression by immunostaining and Western blot analysis.
**Figure S3:** Endothelial cell‐specific Matrin‐3 deficiency doesn't affect body weight, glucose levels in glucose tolerance test and insulin tolerance test in mice fed on a high‐fat diet.
**Figure S4:** Endothelial cell‐specific Matrin‐3 deficiency accelerates the formation of atherosclerotic lesions.
**Figure S5:** Endothelial cell‐specific Matrin‐3 deficiency alters the transcriptome in the aortic intima.

## Data Availability

All data underlying this study are available within the article and supplemental materials. The bulk and single‐nucleus RNA‐seq data have been deposited at the National Center for Biotechnology Information Gene Expression Omnibus (NCBI GEO) under accession numbers GSE335387, GSE335388, GSE335389. Data will be made available upon reasonable request.
