# Supplementary material for: Endothelial Matrin‐3 Limits Cellular Senescence and Lipid Dysregulation to Protect Against Murine Atherosclerosis
Source: FASEB J. 2026 Oct 17;40(20):e72396. doi: 10.1096/fj.202602809RR (PMC13675935; doi:10.1096/fj.202602809RR)
Supplement: Supplementary file 1 [file FSB2-40-e72396-s001.pdf]

Supplemental Data

RESEARCH ARTICLE

Endothelial Matrin-3 Limits Cellular Senescence and Lipid Dysregulation to Protect Against Murine Atherosclerosis

Xiao Cheng[1], Fatema Yeasmin Tanni[1], Rachelle Nelson Zoni[1], Elham Abdollahi[1], Eli Childs[1], Xinghui Sun[1, 3, *]

[1] Department of Biochemistry, University of Nebraska - Lincoln, Beadle Center, 1901 Vine St, Lincoln, Nebraska 68588, USA;

[3] Nebraska Center for the Prevention of Obesity Diseases through Dietary Molecules, University of Nebraska - Lincoln.

* Correspondence: Dr. Xinghui Sun, Department of Biochemistry, Beadle N158, University of Nebraska – Lincoln, 1901 Vine Street, Lincoln, NE 68588-0665, USA. Tel: [+1 402 472 8898], Email: xsun17@unl.edu

Supplemental Figures

Figure S1. Matrin-3 is a protein binding partner of long noncoding RNA Meg3.

Figure S2. Matrin-3 expression by immunostaining and Western blot analysis.

Figure S3. Endothelial cell-specific Matrin-3 deficiency doesn't affect body weight, glucose levels in glucose tolerance test and insulin tolerance test in mice fed on a high-fat diet.

Figure S4. Endothelial cell-specific Matrin-3 deficiency accelerates the formation of atherosclerotic lesions.

Figure S5. Endothelial cell-specific Matrin-3 deficiency alters the transcriptome in the aortic intima.

Figure S1

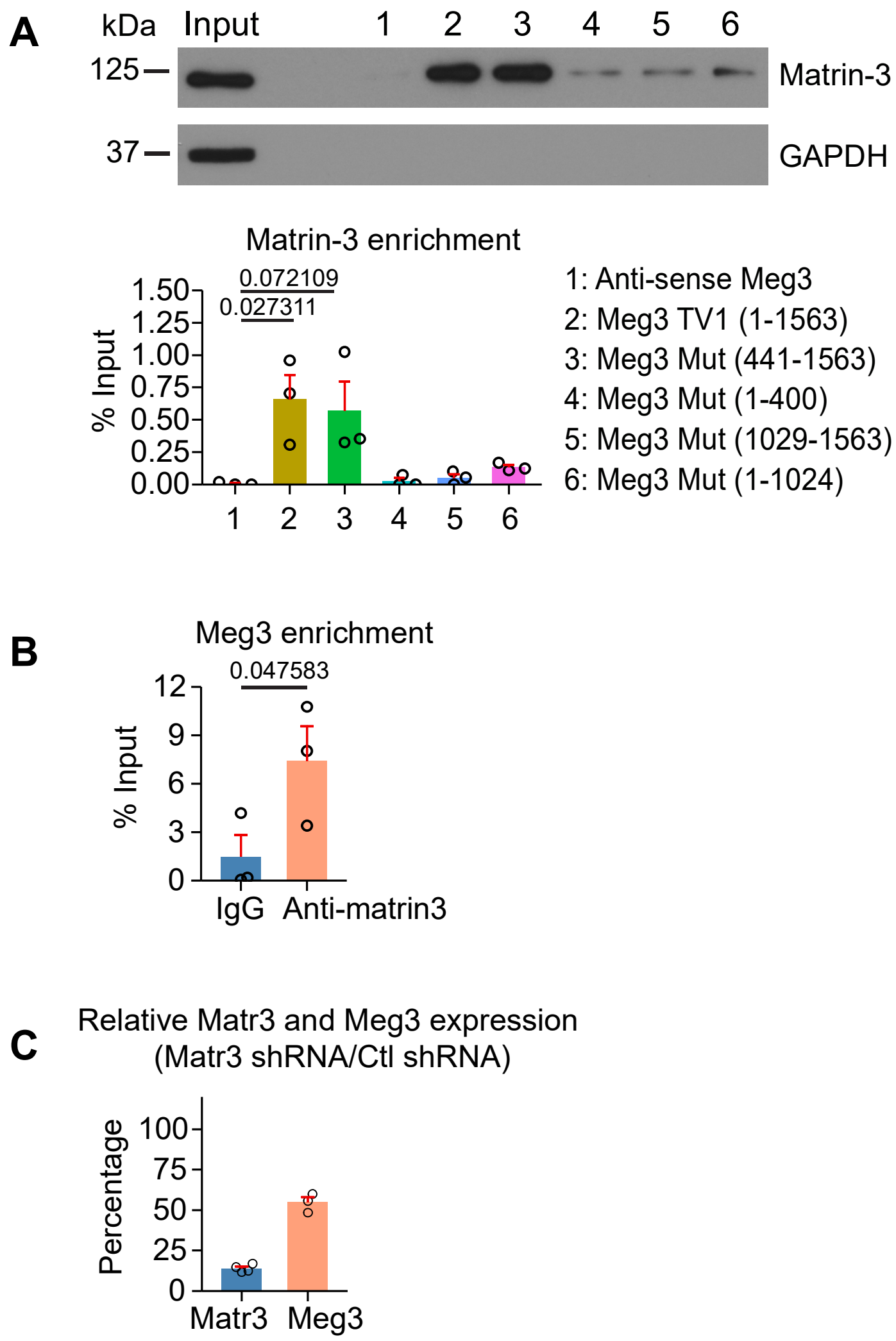

Figure S1. Matrin-3 is a protein binding partner of long noncoding RNA Meg3.

A, The RNA-protein complexes from lncRNA pulldown using antisense Meg3 (negative control RNA), sense Meg3 (TV1), and Meg3 deletion mutants were subjected to western blot analysis of Matrin-3 and GAPDH after separation on SDS-PAGE gel. GAPDH was examined as a negative control protein.

B, The interaction of endogenous Matrin-3 with Meg3 was detected by RNA immunoprecipitation. EC lysates were immunoprecipitated with anti-Matrin-3 antibody or Isotype matched control IgG. Meg3 was examined by qPCR in the immuno-precipitates.

C, The plot shows the percentage of Matrin-3 expression and Meg3 expression in HUVECs infected with Matrin-3 shRNA relative to HUVECs infected with lentiviral control shRNA.

Figure S2

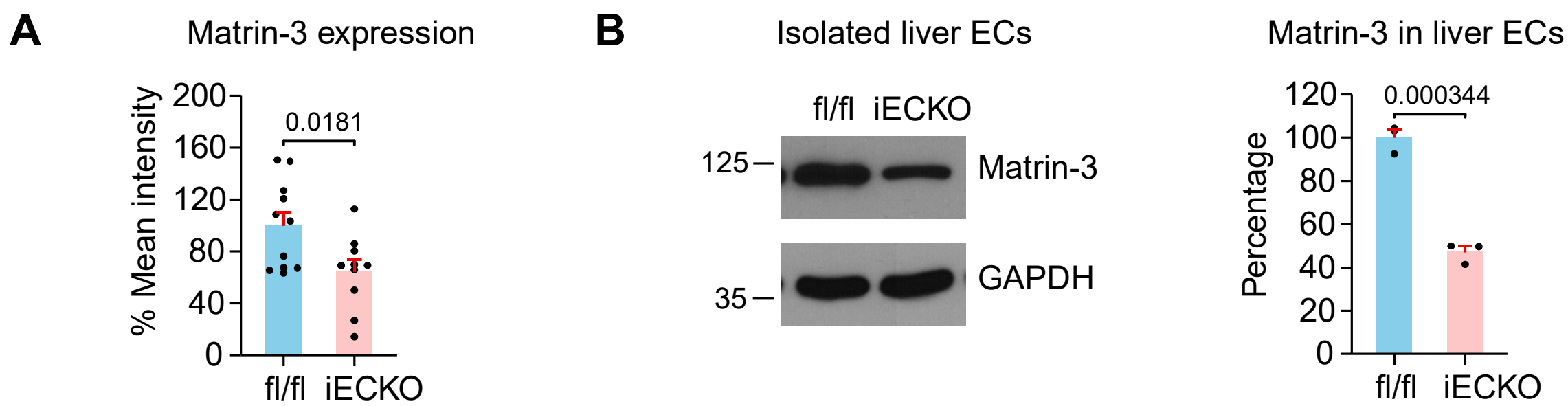

Figure S2. Matrin-3 expression by immunostaining and Western blot analysis.

A, Quantification of Matrin-3 expression by immunostaining in aortic root endothelial cells.

B, Western blot analysis of Matrin-3 in liver ECs isolated from *Matr3* fl/fl and iECKO mice four weeks after tamoxifen administration. Each point represents the data of ECs pooled from two livers.

Figure S3

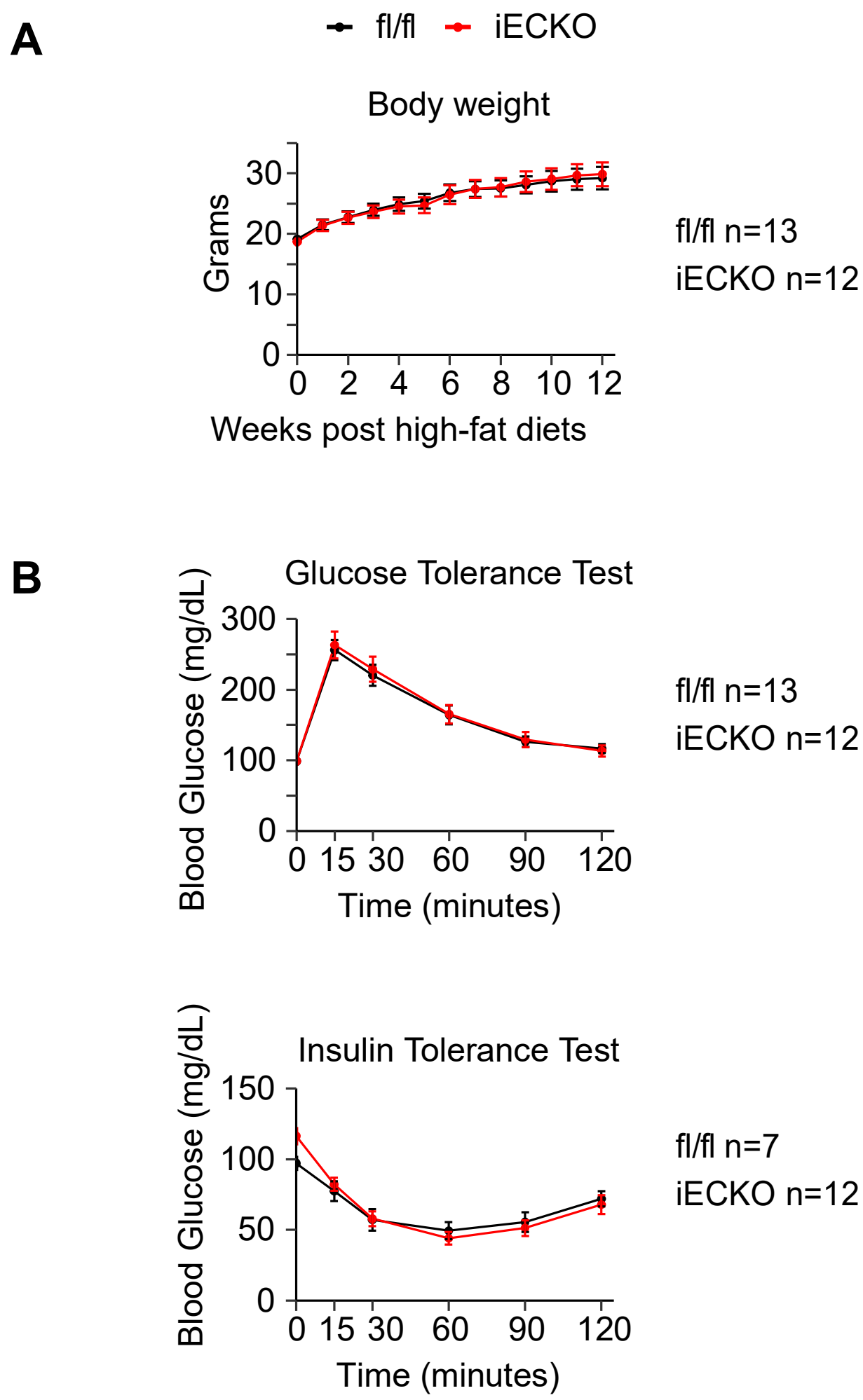

Figure S3. Endothelial cell-specific Matrin-3 deficiency doesn't affect body weight, glucose levels in glucose tolerance test and insulin tolerance test in mice fed on a high-fat diet.

A, Body weights.

B, Glucose levels in glucose tolerance test and insulin tolerance test.

Figure S4

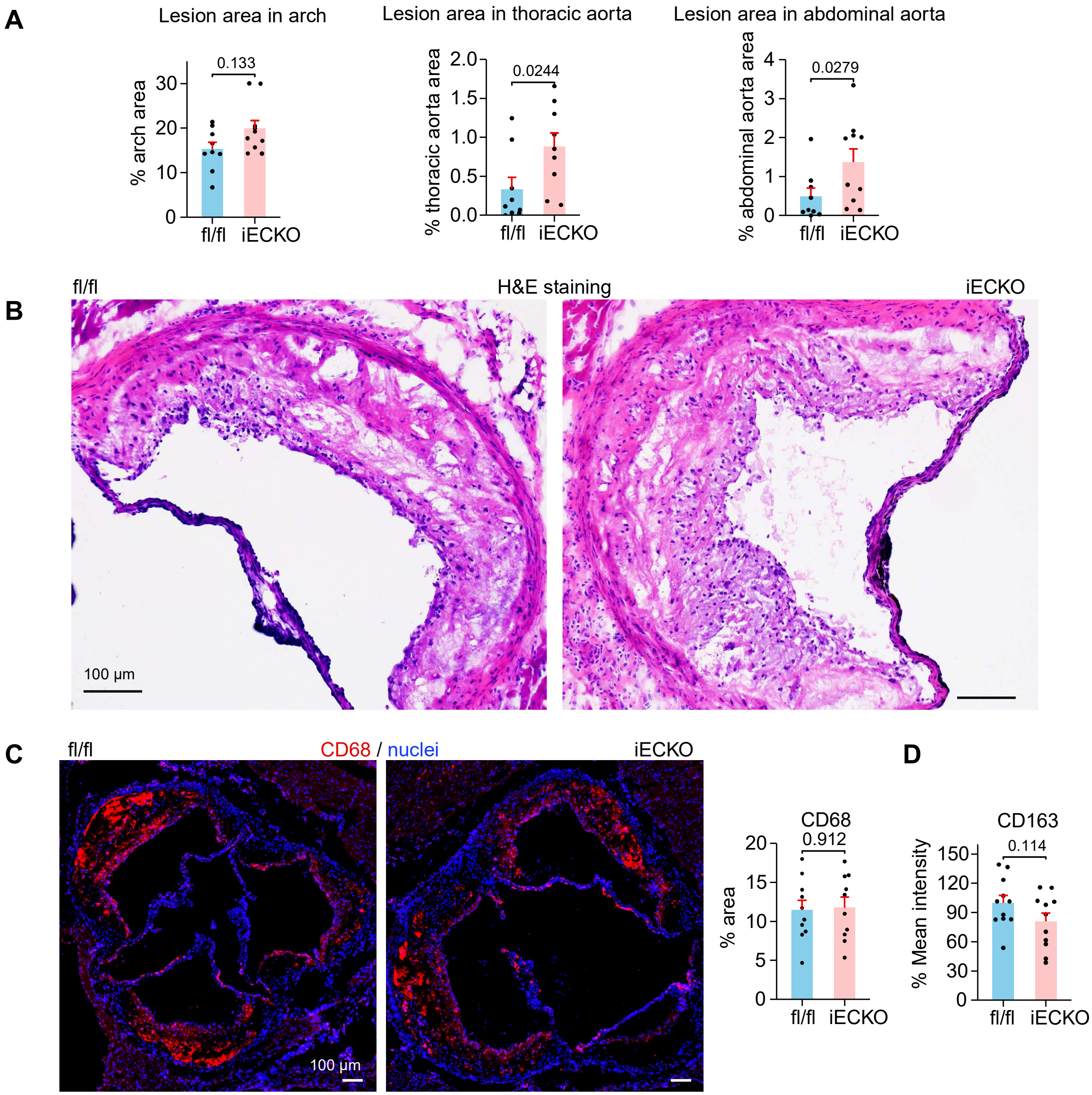

Figure S4. Endothelial cell-specific Matrin-3 deficiency accelerates the formation of atherosclerotic lesions.

A, Quantification of Oil Red O-positive areas in aortic arch, thoracic aorta, and abdominal aorta. The aortas were segmented into arch, descending thoracic aorta, and abdominal aorta three regions for the quantification of lesion area percentages. The lesion area percentage in the whole aorta was also calculated.

B, H&E staining of aortic root sections.

C, CD68 and nuclei (DAPI) immunostaining on the aortic root sections. Images from 2 - 3 valves per mouse sample were used for quantification. The CD68-positive area was normalized by the total lesion area.

D, CD163 immunostaining of aortic root sections.

Figure S5

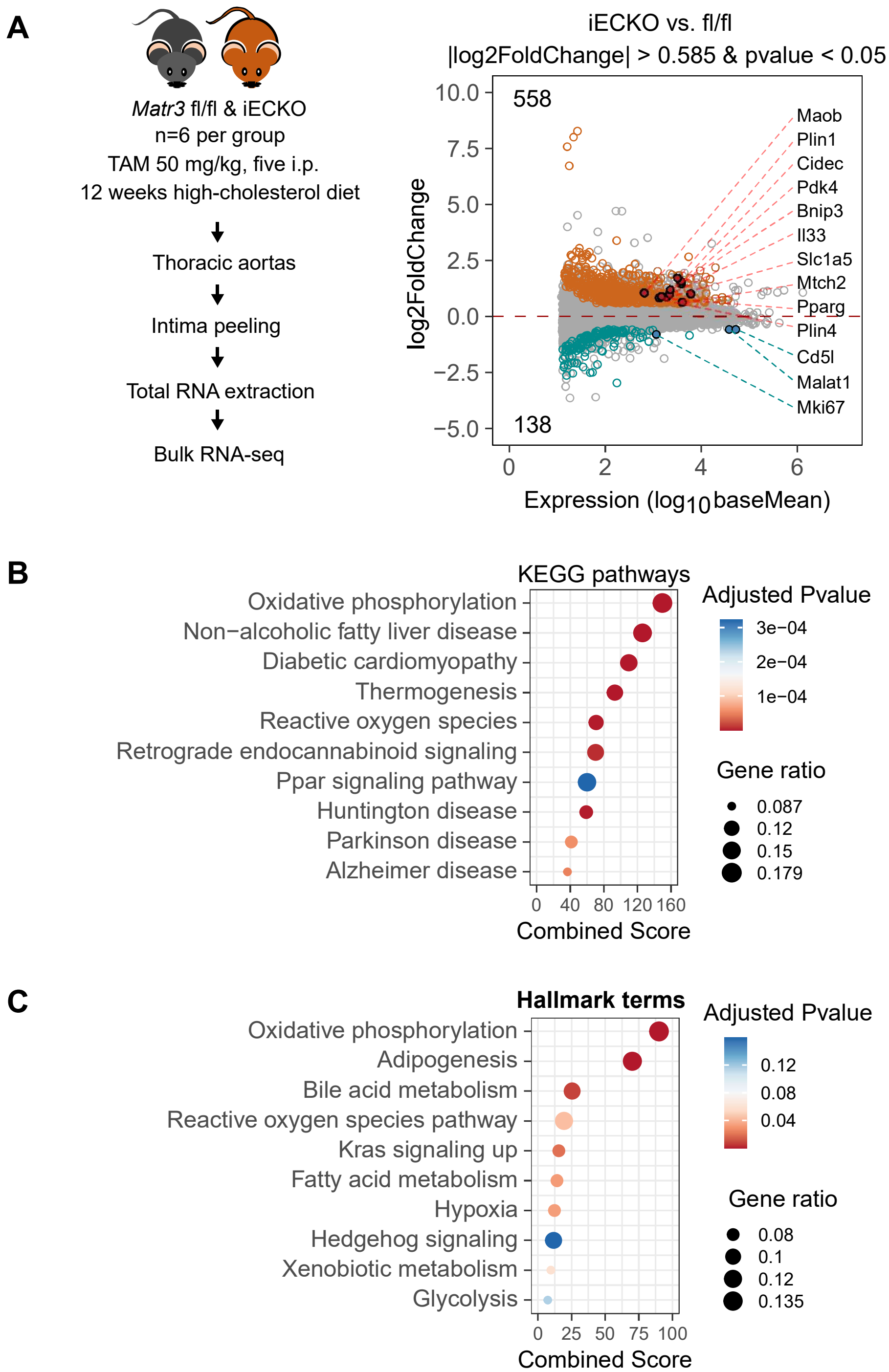

Figure S5. Endothelial cell-specific Matrin-3 deficiency alters the transcriptome in the aortic intima.

A, Schematic depicting the workflow and a MA plot showing the differentially expressed genes identified by bulk RNA-seq.

B, Top KEGG pathways identified by Enrichr analysis. 696 differential genes (|log2FoldChange| > 0.585; padj < 0.05) were used for analysis.

C, Top Hallmark terms identified by Enrichr analysis. 696 differential genes (|log2FoldChange| > 0.585; padj < 0.05) were used for analysis.
